# Recognition‐Encoded Molecules: A Minimal Self‐Replicator

**DOI:** 10.1002/chem.202401667

**Published:** 2024-10-31

**Authors:** Daniele Rosa‐Gastaldo, Francesco Maria Ara, Andrea Dalla Valle, Giulia Visentin, Luca Gabrielli

**Affiliations:** ^1^ Department of Chemical Sciences University of Padova via Marzolo 1 35131 Padova (PD) Italy

**Keywords:** Self-replicator, Oligoaniline, Duplex, Recognition, H-bonding

## Abstract

Nucleic acids, with their unique duplex structure, which is key for information replication, have sparked interest in self‐replication's role in life's origins. Early template‐based replicators, initially built on short oligonucleotides, expanded to include peptides and synthetic molecules. We explore here the potential of a class of synthetic duplex‐forming oligoanilines, as self‐replicators. We have recently developed oligoanilines equipped with 2‐trifluoromethylphenol–phosphine oxide H‐bond base pairs and we investigate whether the imine formed between aniline and aldehyde complementary monomers can self‐replicate. Despite lacking a clear sigmoidal kinetic profile, control experiments with a methylated donor and a competitive inhibitor support self‐replication. Further investigations with the reduced aniline dimer demonstrate templated synthesis, revealing a characteristic parabolic growth. After showing sequence selective duplex formation, templated synthesis and the emergence of catalytic function, the self‐replication behaviour further suggests that the unique properties of nucleic acids can be paralleled by synthetic recognition‐encoded molecules.

## Graphical abstract

A minimal donor‐acceptor heterodimer, based on hydrogen bonding recognition, exhibits self‐replicating behaviour through imine formation between aniline and aldehyde complementary monomers. This further suggests that the unique properties of nucleic acids can be paralleled by synthetic recognition‐encoded molecules.

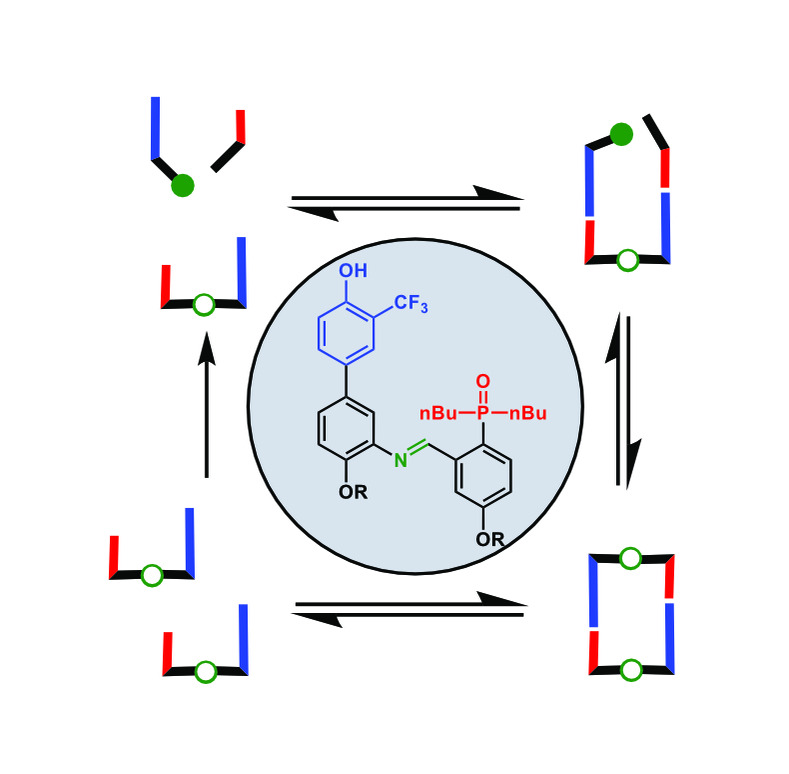

## Introduction

Nucleic acids possess unique properties since the duplex architecture allows them to replicate, transduce, and translate information that are encoded as a sequence of recognition‐encoded monomers. In particular, self‐replication has attracted attention because of the key role it played in the emergence of life.[^1^, ^2^, ^3^] Hence, the first non‐enzymatic template‐based replicators were based on a short palindromic oligonucleotide associated into a duplex, which acted as the template for the formation of a third strand from two fragments.[^2^, ^3^, ^4^] Subsequently, self‐replicators have been developed using peptides[^5^, ^6^, ^7^, ^8^, ^9^, ^10^, ^11^, ^12^, ^13^, ^14^, ^15^, ^16^, ^17^, ^18^, ^19^, ^20^, ^21^, ^22^] and synthetic small molecules.[^23^, ^24^, ^25^, ^26^, ^27^, ^28^, ^29^, ^30^, ^31^, ^32^, ^33^, ^34^, ^35^, ^36^]

Reasoning that synthetic recognition‐encoded oligomers have the potential to display similar properties to nucleic acids, scientists first used nucleic acid derivatives[^37^, ^38^, ^39^, ^40^, ^41^, ^42^, ^43^, ^44^, ^45^, ^46^, ^47^, ^48^, ^49^, ^50^, ^51^, ^52^, ^53^, ^54^] and then developed a range of sequence‐selective duplex forming oligomers.[^55^, ^56^, ^57^, ^58^, ^59^, ^60^, ^61^, ^62^, ^63^, ^64^, ^65^, ^66^, ^67^, ^68^, ^69^, ^70^, ^71^] We have recently developed a class of duplex forming oligoanilines based on the H‐bond base pair 2‐trifluoromethylphenol–phosphine oxide and an aniline backbone. We indeed observed that this class of molecules can have properties that parallel those of nucleic acids, such as polymerase activity,^[72]^ sequence selective duplex formation and the hydrogen bond driven template effect.[^73^, ^74^] Here we want to explore whether a minimal hydrogen bonding heterodimer can function as a self‐replicant.

**Figure 1:**
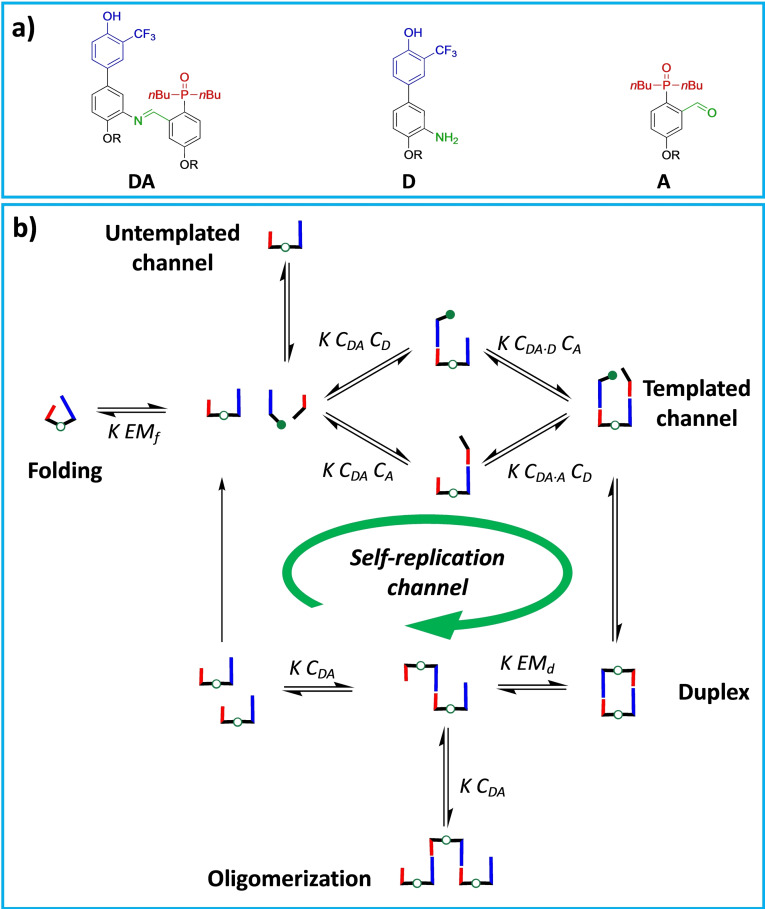
a) Chemical structure of the recognition encoded molecules that compose the system: the monomers are equipped with hydrogen bond donor (trifluoromethylphenol, blue, for the aniline **D**) and acceptor (phosphine oxide, red, for the aldehyde **A**). Imine heterodimer **DA** is shown, and the linker chemistry is highlighted in green. R = 2‐ethylhexyl. b) Cartoon showing the pathways involved in this minimal replicating system: the imine template can be formed through a non‐templated channel and *via* self‐replication. The self‐assembly channels involving the imine dimer **DA** are highlighted: the first base‐pairing interaction can take place in an intramolecular fashion, leading to the 1,2‐folding path, or intermolecularly with a **DA** dimer or **D/A** monomers, leading respectively to the duplex channel or supramolecular oligomerization (template inhibition), or to the trimeric complex (templated channel). The outcome depends on the concentrations of the components *C*, the association constant for the intermolecular base‐pairing interaction *K*, and the effective molarities for folding (*EM_f_*) and duplex formation (*EM_d_*).

In fact, the reaction between donor aniline (**D**) and the aldehyde acceptor (**A**) monomers is a potential self‐replicator system since the formed **DA** imine dimer can act as a template (Figure 1a).

The imine dimer **DA** can be formed *via* both untemplated and templated pathways. Once the **DA** imine dimer appears in solution, the key ternary complex **DA ⋅ D ⋅ A** is formed, triggering an autocatalytic cycle. This should lead to an exponential increase of [**DA**], with the concentration‐time profile ideally following a sigmoidal curve. However, it is well documented that some experimental systems do not display a sigmoidal shape, which becomes visible only above critical values of autocatalytic efficiency ϵ, defined as the ratio of the template‐catalysed to the template‐independent rate constants.[^75^, ^76^, ^77^] Additionally, in templated self‐replicators product inhibition arises due to the dimerization of the template, rendering it catalytically inactive (Figure 1b). Consequently, these systems display parabolic growth, and their behaviour was firstly described by von Kiedrowski with the empirical square root law of autocatalysis, where the autocatalytic reaction order is not 1, but 1/2.^[2]^

In this paper we will demonstrate that the system based on the minimal heterodimer **DA** is behaving as a self‐replicator. Similarly to oligonucleotide palindromic sequences, the system suffers from product inhibition, which leads to parabolic growth.

## Results and Discussion

We initially reasoned about what conditions might allow the system to behave as a replicating. When concentrations of components are constant, the self‐assembly behaviour of a mixture of monomers (**D**, **A**) and the corresponding imine dimer (**DA**) is determined by the interplay of different key parameters (Figure 1b): the concentration of monomers (*C_D_* and *C_A_*) and imine (*C_DA_*), the association constant for intermolecular base‐pairing interaction *K* and the effective molarities for duplex formation (*EM_d_*) and folding (*EM_f_*).

As shown in Figure 1b, the channel for templated reaction competes with the folding pathway, the duplex formation, and the network formation channels. Notably, intramolecular interaction between adjacent units is the most detrimental, as the intramolecular process will dominate over both the duplex and the templated pathways. However, we have previously studied^[73]^ the self‐assembly properties of monomers **D**, **A** and dimer **DA** in chloroform (provided for convenience in Table 1). We demonstrated that in the **DA** dimer the 1,2‐folding between complementary units is absent (*EM_f_* = 0),^[73]^ and this is important because it indicates the availability of recognition modules for intermolecular binding.

**Table 1:** Association constants (*K*, [M^−1^]) expressed as log*K*, effective molarities (*EM*) and population of folded dimer in the monomeric state measured by ^1^H and ^19^F‐NMR titrations in chloroform‐*d* at 298 K.^[a][73]^

Complex	log *K_obs_* (M^−1^)	*EM*_f_ (mM)	*X*_fold_
D•A	2.4±0.1	–	–
DA•DA	3.3±0.1	0.0	0.0

^[a]^ Each titration was repeated twice, and the average value is reported with errors at the 95 % confidence limit.

Thus, the competitors against the formation of the key trimeric complex **DA ⋅ D ⋅ A** are the duplex, and the network formation pathways, eventually resulting in template deactivation. However, intermolecular networks only become significant at dimer concentrations higher than the effective molarity for duplex formation (around 50 mM, assuming that **DA ⋅ DA** and **DD ⋅ AA** duplexes have similar *EM_d_*).^[73]^

Therefore, for product concentrations lower than 50 mM, the main competitor of the templated channel is duplex formation. We can reasonably assume that the formation of the **DA ⋅ D ⋅ A** complex stems from two independent binding events on the dimer **DA**. Thus, given that the association constant (*K*) for the intermolecular base‐pairing interaction is around 250 M^−1^ in chloroform,^[73]^ we can describe the overall formation of the trimeric complex **DA ⋅ D ⋅ A** as shown in equation 1:
(1)

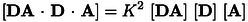




**Figure 2:**
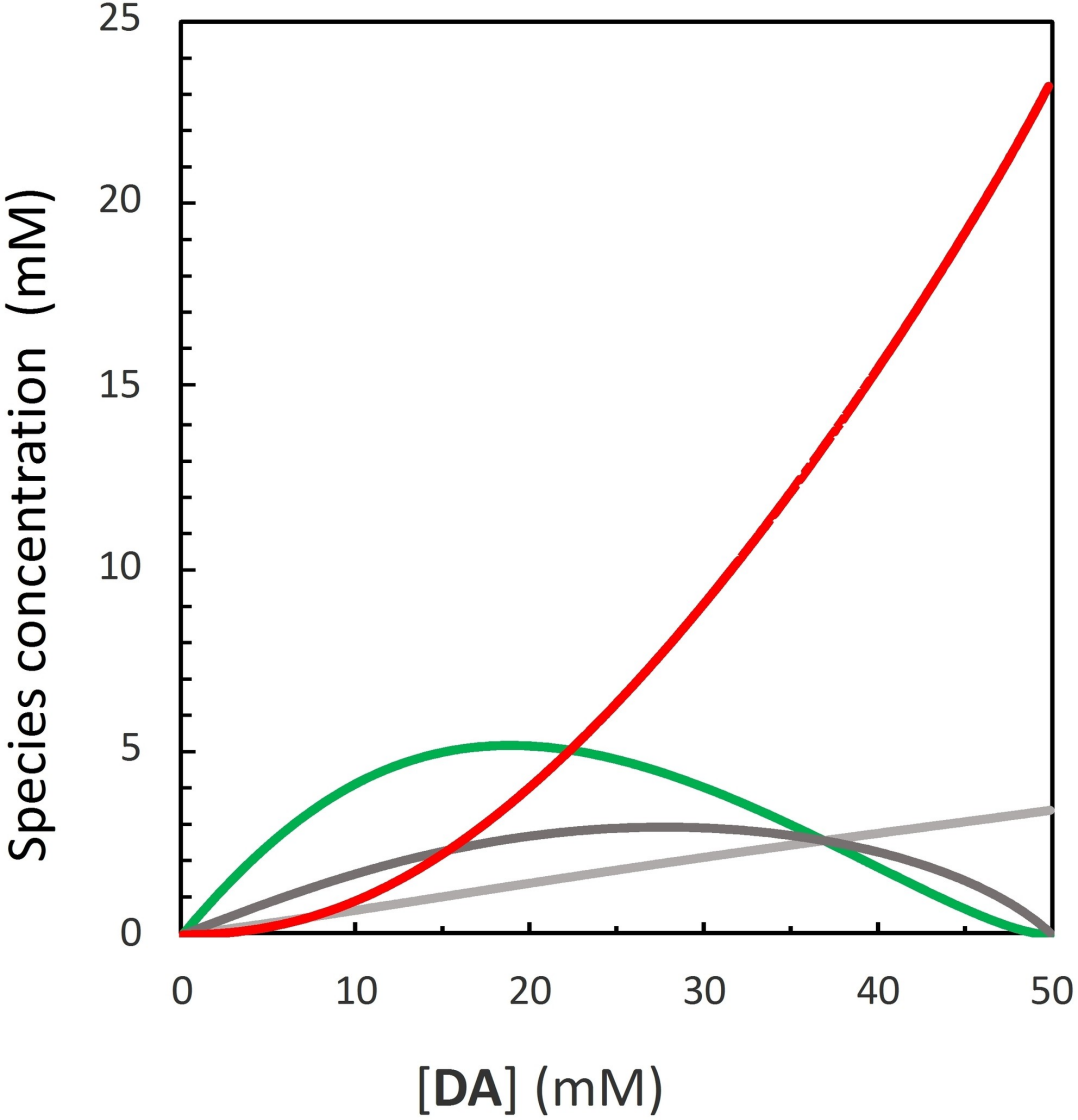
a) Concentration of self‐assembled **DA** species for increasing concentration of imine formed in a chloroform solution of aniline **D** and aldehyde **A**, 50 mM each. Trimeric complex [**DA ⋅ D ⋅ A**] is shown in green, duplex [**DA ⋅ DA**] in red, complexes [**DA ⋅ D**] and [**DA ⋅ A**] in dark grey and [free **DA**] in light grey.

Considering that the observed association constant (*K_obs_*) for self‐dimerization of **DA** is around 2000 M^−1^ in chloroform, we computed the population of **DA**‐containing species for a solution of monomers **D** and **A** ranging from 50 to 0 mM, with consequent **DA** imine increase from 0 to 50 mM, while disregarding the contribution of intermolecular networks (Figure 2).

**Figure 3:**
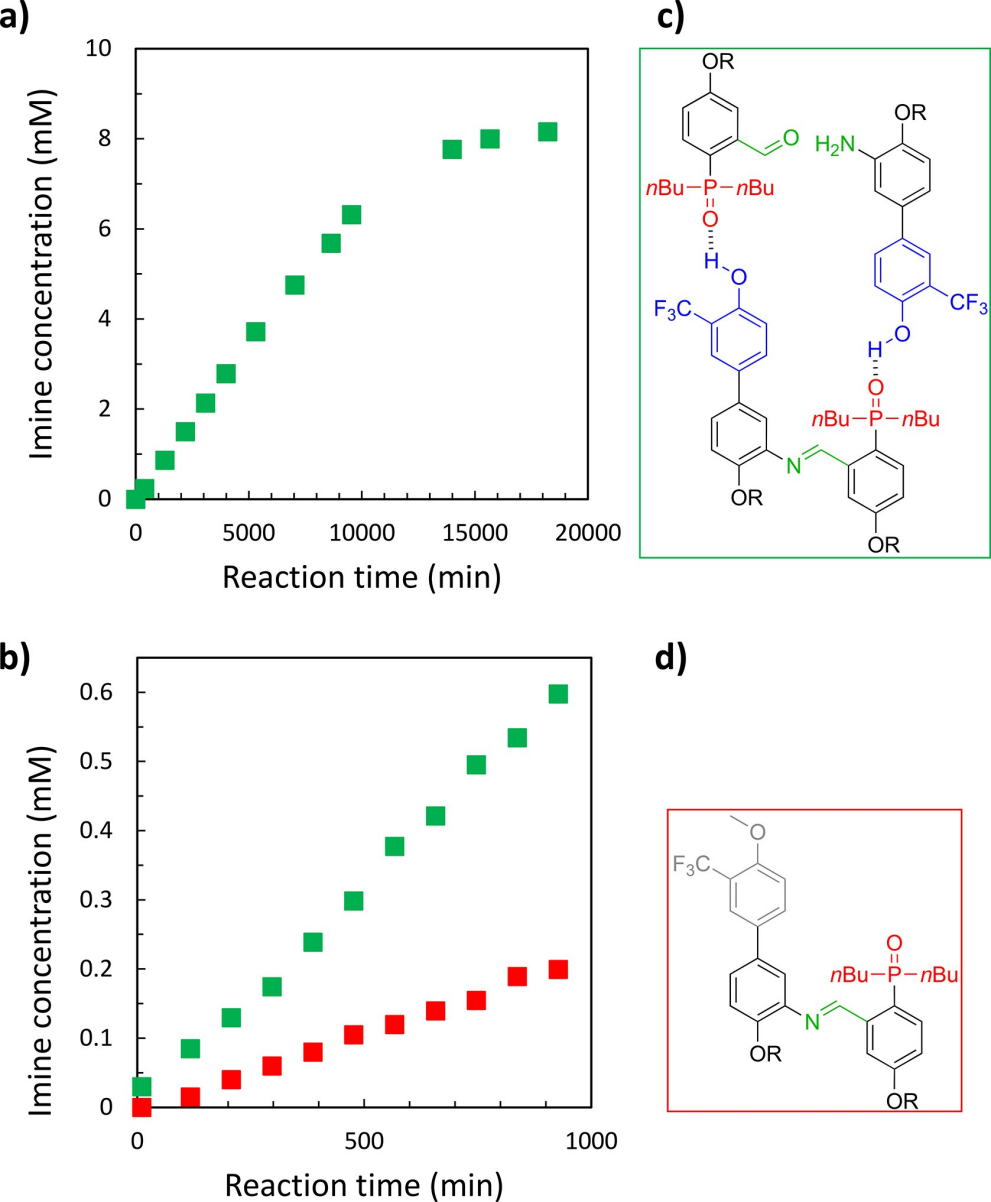
a) Imine formation in chloroform‐*d* at 298 K between **D** and **A** (50 mM each in a well‐sealed tube). b) Imine formation in chloroform‐*d* at 298 K between **D** and **A** (50 mM each, green squares) and **D^OMe^** and **A** (50 mM each, red squares). The best fit of the data is reported in Figure S2.3 and Figure S2.5 in the SI. c) Structures of the **DA ⋅ D ⋅ A** trimeric complex (green square, top) and d) **D^OMe^A** imine (red square, bottom). R = 2‐ethylhexyl.

The concentration of monomers must be sufficiently high to allow the formation of the complex **DA ⋅ D ⋅ A**, yet low enough to minimize the non‐templated formation of imine **DA**. Figure 2 shows that in the early stages of a 50 mM reaction in chloroform, the generated imine dimer mainly assembles into the trimeric complex **DA ⋅ D ⋅ A**, responsible for the potential templated formation of imine. When the concentration of imine product reaches 19 mM, the percentage of **DA** assembled into duplex **DA ⋅ DA** matches that of the dimer assembled into the trimeric complex. As the reaction progresses, the increasing dimer concentration leads to enhanced duplex formation and a decay in the trimeric complex, indicating stronger product inhibition. Since these preliminary investigations suggested that the system can behave as a self‐replicant under specific conditions, we decided to study via ^1^H‐NMR the imine formation in a 50 mM solution of aniline **D** and aldehyde **A** in chloroform‐*d*. Figure 3a (see SI section 2) shows that the reaction reaches the equilibrium after around 10 days, with an imine percentage close to 17 %. Notably, the kinetic profile does not show a clear sigmoid that could be expected for a self‐replicant system. However, since the kinetic profile exhibits a sigmoidal shape only above critical values of autocatalytic efficiency ϵ,^[75]^ we proceeded with different control experiments to evaluate the self‐replication potential of the **DA** system.^[76]^

We therefore used the methylated donor monomer (**D^OMe^**, Figure 3d) as a control compound, which shares the same chemical functionality as monomer **D**, but lacks the ability to engage in the H‐bonding recognition process necessary for the self‐replication. Comparison between the methylated and the non‐methylated systems allows us to evaluate the ability of the **DA** imine to template its own formation. Figure 3b shows that the use of **D^OMe^** slows down imine formation kinetics, with the initial reaction rate decreasing from 0.64 to 0.22 μM min^−1^.

**Figure 4:**
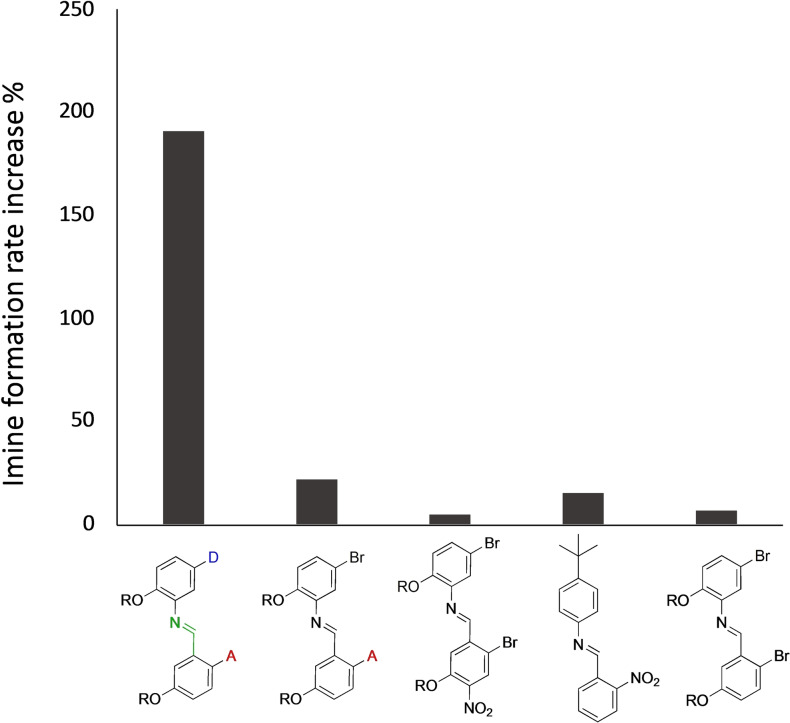
Percentage of imine formation rate increase caused by the presence of an equimolar amount of phenol. The kinetic experiments were performed as detailed in the section S1.1 of the SI. A detail of the structures of the involved anilines and aldehydes is reported in Scheme S1.1. The formation rate of **DA** was compared with the one of **D^OMe^A**. For the other imines, 50 mM of trioctylphosphine oxide (0 mM when using aldehyde **A**) and 0 mM (no‐phenol control experiment) or 50 mM of 4‐bromo‐2‐(trifluoromethyl)phenol (phenol experiment) were added into the solutions. See section 3 of the SI. R = 2‐ethylhexyl.

Since we previously observed that the phenol can catalyze the condensation between simple anilines and benzaldehydes lacking any recognition group,^[72]^ we designed control experiments to assess the presence and magnitude of a phenol‐catalyzed imine formation channel (Figure 4 and SI section 3). The effects of a phenol unable to form imines, were assessed on the acceptor aldehyde **A** and on a set of benzaldehydes derivatives lacking the phosphine oxide recognition unit. In the formation of imine **DA** the phenol unit is in the presence of an equimolar amount of phosphine oxide acceptor, thus implying that the amount of free phenol is strongly reduced. Hence, when the phosphine oxide was not directly attached to the aldehyde monomer, an equimolar amount of trioctylphosphine oxide was added to the reaction mixture. We therefore studied *via* ^1^H‐NMR the imine formation in 50 mM chloroform‐*d* solutions of anilines and aldehydes, in the presence and in absence of an equimolar amount of a trifluoromethyl phenol unable to form imines. The percentage of the imine formation rate increase caused by the presence of phenol is plotted for **DA** and four different control imines in Figure 4. The increase in reaction rate purely caused by phenol catalysis ranges between 5 and 20 %, which is significantly lower than the rate increase observed when comparing **D^OMe^A** and **DA** (190 %).

**Figure 5:**
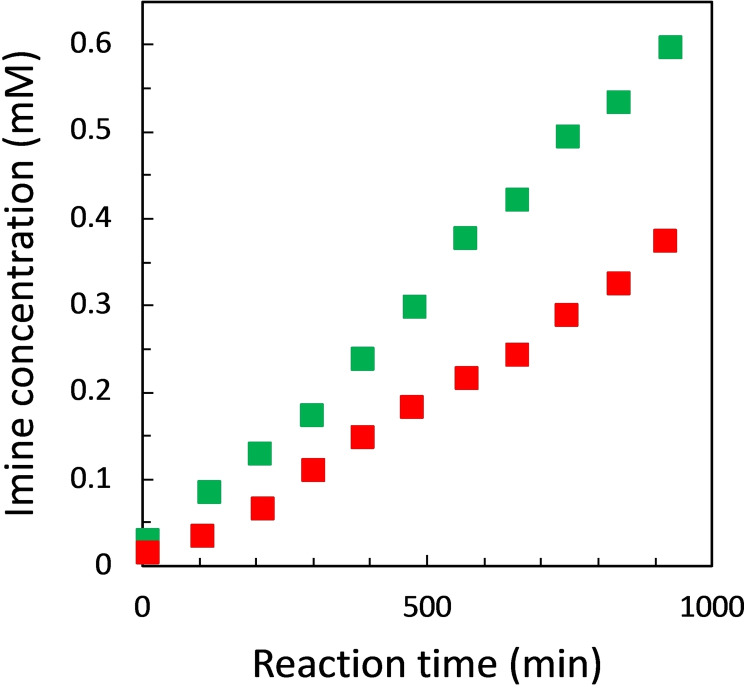
a) Imine formation in chloroform‐*d* at 298 K between **D** and **A** (50 mM each in air‐tight tube) without (green squares) and in the presence of trioctylphosphine oxide (50 mM, red squares). The best fit of the data is reported in Figure S2.7.

Next, to demonstrate that the functioning of the self‐replicator relies on reversible binding between complementary recognition units, we performed self‐replication experiments in the presence of one equivalent of trioctylphosphine oxide as a competitive inhibitor. The phosphine oxide, although unreactive, can bind the donor recognition sites present in **D** and in the imine **DA**, thus interfering with the recognition process. Furthermore, we have previously shown that trialkylphosphine oxides do not affect imine formation between aniline and benzaldehyde lacking recognition units.^[72]^ The results of ^1^H‐NMR kinetic experiments are shown in Figure 5 (SI Section 2). The reaction rate between **D** and **A** is significantly slower in the presence of the inhibitor: one equivalent of trioctylphosphine oxide reduces *V_init_* from 0.64 to 0.40 μM min^−1^. These results confirm that H‐bond interactions between acceptor and donor recognition units play an important role in the self‐replication process.

**Figure 6:**
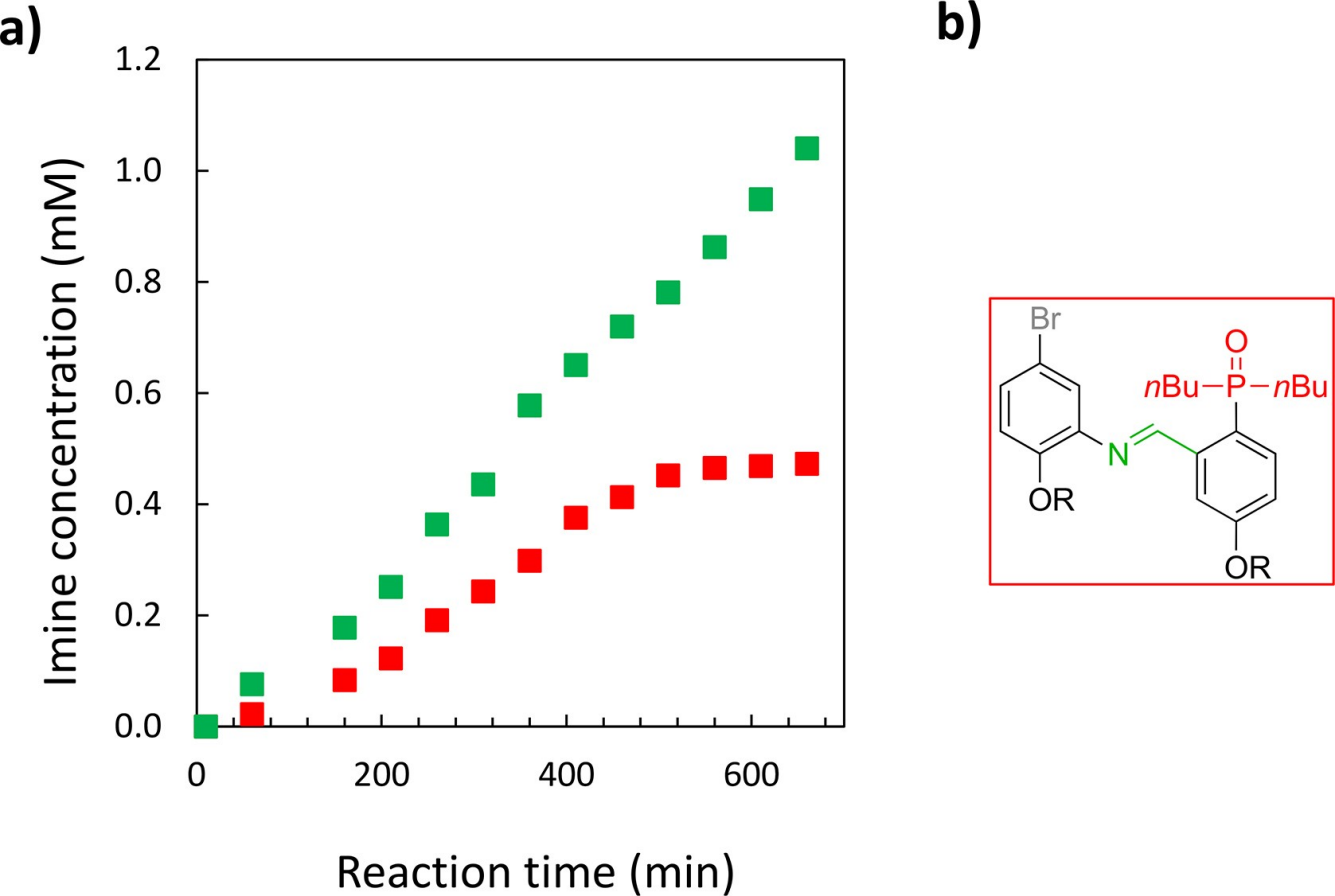
a) Imines formation at 298 K in a chloroform‐*d* solution containing aldehyde **A** (50 mM) and anilines **D** and **Br_NH2_** (50 mM each). Imine **DA** is represented with green squares, imine **Br_NH2_A** with red squares. b) Structure of **Br_NH2_A** imine (R = 2‐ethylhexyl).

To further exploit the importance of the recognition process, we decided to study *via* ^1^H‐NMR the imine formation in an equimolar solution of aldehyde **A** and anilines **D** and **Br_NH2_** (50 mM each, SI section 4). Figure 6a shows that the presence of the recognition has two distinct effects. The first is kinetic and it is related to the preorganization of the monomers on the template that facilitates the imine formation, which is two times faster for **DA** than for **Br_NH2_A** (chemical structure in Figure 6b). On the other hand, the reversibility of the imine linker allows a second effect, which is thermodynamic, and it is associated with an increased product concentration at the equilibrium. In fact, **DA** equilibrates around 8 mM (see Figure 3a), while the formation of **Br_NH2_A** imine, which is unable to self‐assemble into dimers, reaches a plateau around 0.5 mM.

The final experiment aimed at providing evidence of self‐replication within the **DA** system, involved doping the reaction with different amounts of the template product itself. However, as the system relies on the formation of a reversible covalent bond, the addition of the imine product can sensibly reduce the amount of formed dimer.

**Figure 7:**
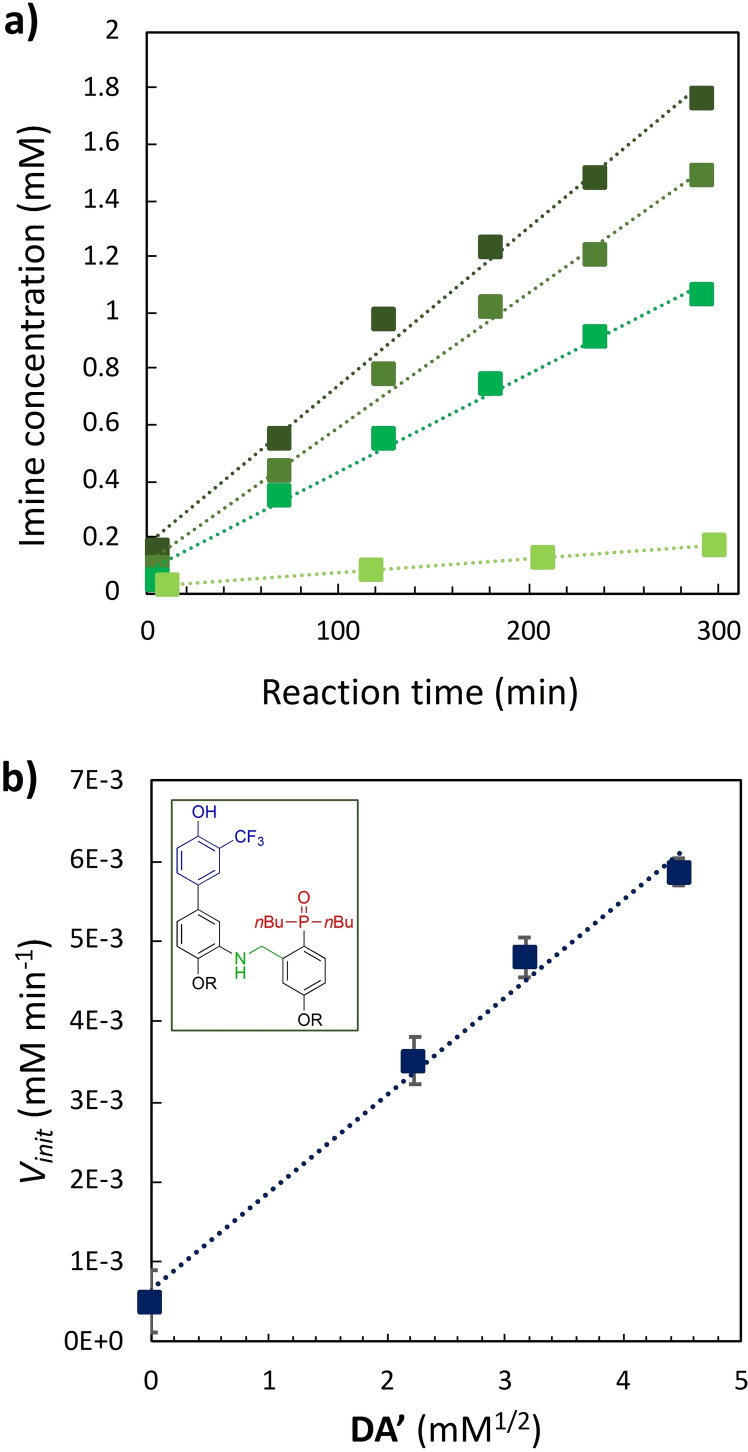
a) Imine formation in chloroform‐*d* at 298 K between **D** and **A** (50 mM each in a well‐sealed tube) in the presence of aniline template **DA’** (0, 5, 10, 20 mM, respectively from light to dark green) *V_init_* data, as result of the best fit line reported, are summarized in Table S2.1 in the SI. b) The initial reaction rate (*V*_init_) of imine formation between **D** and **A** (50 mM each) is plotted as a function of the square root of **DA’** concentration. The line of best fit shown is *V_init_* =1.22 ⋅ 10^−3^ [**DA’**]^1/2^+6.46 ⋅ 10^−4^. According to equation (2) a=1.22 ⋅ 10^−3^ mM^1/2^ min^−1^ and b=6.46 ⋅ 10^−4^ mM min^−1^ (respectively 6.19 ⋅ 10^−7^ M^1/2^ s^−1^ and 1.16 ⋅ 10^−8^ M s^−1^). The structure of aniline **DA’** is shown in the insert.

This issue has been previously solved by using the reduced amine as template molecule.^[78]^ Hence, equimolar chloroform solutions of **D** and **A** (50 mM each) were subjected to reaction in the presence of varying amounts of the aniline template dimer **DA’** (ranging from 0 to 20 mM, Figure 7a, SI section 2) and the progress of the reactions was monitored via ^1^H‐NMR. For all tested conditions, initial rates of template synthesis *V_init_*, were determined from concentration–time profiles at early reaction times and plotted as a function of the square root of the template concentration (Figure 7b).

The plot shown in Figure 7b reveals a linear correlation between *V_init_* and the square root of the aniline template concentration [**DA’**]. This allows to establish the empirical relationship (2), indicating that the complementary imine is formed from monomers **D** and **A** via two pathways: a template‐catalysed route, characterized by the empirical constant *a*, and a template‐independent bimolecular process measured with the empirical constant *b*, representing the initial rate in the absence of **DA’**.
(2)

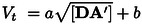




The rate of templated synthesis follows the square‐root of the initial template concentration. This peculiar rate law is typical of self‐replicant systems afflicted by product inhibition, where the product remains bound to the template, causing its inactivation. This behaviour is described as parabolic growth.[^2^, ^4^, ^79^] The ratio between templated and non‐templated reaction rates is defined as the autocatalytic efficiency (ϵ), which for this system is 59 M^−1/2^, that is higher, but still comparable to the ϵ obtained with similar imine‐based replicants of Von Kiedrowski (16 M^−1/2^). Furthermore, this value explains why the kinetic profile did not show the sigmoidal profile, which only becomes clearly visible with ϵ values higher than 10^2^ for reactions with an autocatalytic order of 1/2.^[75]^

## Conclusions

We studied the behaviour of a minimal self‐replicator based on H‐bonded recognition‐encoded oligoanilines. Previous studies on this class of molecules have shown that the rigid backbone prevents intramolecular folding between adjacent complementary units and the ability to promote templated synthesis of a homodimer. These results prompted us to investigate whether a system comprising donor aniline **D** and acceptor aldehyde **A** monomers could behave as a self‐replicator. Knowing the association constant for intermolecular base‐pairing interaction and for the dimer self‐association, we initially investigated the experimental conditions that would favour the formation of the key ternary complex **DA ⋅ D ⋅ A**. However, following the formation of **DA** imine we observed the absence of the sigmoidal kinetic profile. Thus, we performed different control experiments to demonstrate that self‐replication is involved in the formation of imine **DA**. The imine formation rate was measured via ^1^H‐NMR also using monomers uncapable of H‐bonding recognition and in the presence of an unreactive competitive inhibitor. The contribution of a phenol‐catalysed pathway has been evaluated and resulted to be marginal compared to the self‐replication channel. The importance of the recognition process has been highlighted in a competition experiment between recognition enabled and recognition disabled monomers, which showed that the self‐assembly affects not only the kinetic, but also the thermodynamic of the process, resulting in a higher imine concentration at the equilibrium. Additionally, the ^1^H‐NMR kinetics of reactions doped with varying amounts of the reduced aniline dimer **DA’** showed that the template effectively pre‐organizes the donor monomers, accelerating the complementary imine formation. We found a linear relationship between the initial rate and the square root of the template concentration, indicating that the **DA** imine formation occurs via two pathways: a template‐catalysed route and a template‐independent bimolecular process. The obtained autocatalytic value well explains the absence of a sigmoidal shaped kinetic profile. Due to template self‐inhibition, the studied systems displayed parabolic growth, akin to observations previously reported not only in small molecule systems, but also on nucleic acids based self‐replicators. After demonstrating sequence‐selective duplex formation, templated synthesis and the emergence of catalytic function, the self‐replication of a minimal heterodimer further suggest that the properties of synthetic recognition encoded molecules can parallel those of natural nucleic acids.

## Conflict of Interests

The authors declare no conflict of interest.

1

## Supporting information

As a service to our authors and readers, this journal provides supporting information supplied by the authors. Such materials are peer reviewed and may be re‐organized for online delivery, but are not copy‐edited or typeset. Technical support issues arising from supporting information (other than missing files) should be addressed to the authors.

Supporting Information

## Data Availability

The data that support the findings of this study are available in the supplementary material of this article.
