# Supplementary material for: Recognition‐Encoded Molecules: A Minimal Self‐Replicator
Source: Chemistry. 2024 Oct 31;30(69):e202401667. doi: 10.1002/chem.202401667 (PMC11632402; doi:10.1002/chem.202401667)
Supplement: Supplementary file 1 — Supporting Information [file CHEM-30-e202401667-s001.pdf]

Chemistry–A European Journal

Supporting Information

Recognition-Encoded Molecules: A Minimal Self-Replicator

Daniele Rosa-Gastaldo,* Francesco Maria Ara, Andrea Dalla Valle, Giulia Visentin, and Luca Gabrielli*

Supporting Information

Table of Contents

1. General experimental procedures and synthetic details S2

1.1. Setup of a ^{1}H NMR kinetics S3

1.2. Synthetic procedures S4

2. Complete ^{1}H NMR kinetics spectra S6

2.1. **D** 50 mM + **A** 50 mM S6

2.2. **D^{OMe}** 50 mM + **A** 50 mM S7

2.3. **D** 50 mM + **A** 50 mM + **1** 50 mM S8

2.4. **D** 50 mM + **A** 50 mM + **DA’** variable concentrations S9

3. Evaluation of the phenol role in the catalysis of the imine formation

3.1. **Br$_{CO}$** 50 mM + **Br$_{NH2}$** 50 mM + **1** 50 mM S12

3.2. **Br$_{CO}$** 50 mM + **Br$_{NH2}$** 50 mM + **1** 50 mM + **2** 50 mM S12

3.3. **Br$_{CO}^{NO2}$** 50 mM + **Br$_{NH2}$** 50 mM + **1** 50 mM S13

3.4. **Br$_{CO}^{NO2}$** 50 mM + **Br$_{NH2}$** 50 mM + **1** 50 mM + **2** 50 mM S13

3.5. **3** 50 mM + **4** 50 mM + **1** 50 mM S14

3.6. **3** 50 mM + **4** 50 mM + **1** 50 mM + **2** 50 mM S14

3.7. **A** 50 mM + **Br$_{NH2}$** 50 mM S15

3.8. **A** 50 mM + **Br$_{NH2}$** 50 mM + **2** 50 mM S15

4. Competitive experiment

4.1. Competitive experiment S16

1. General experimental procedures and synthetic details

The solvents used in the purification and characterization steps were bought from international suppliers and used without prior purification. All the molecules involved in the study (**D**, **A**, **DA'**, **D^{OMe}**, **Br$_{NH2}$**, **Br$_{CO}$**, **Br$_{CO}^{NO2}$**, Scheme S1.1) were synthesised as reported in Rosa-Gastaldo et al. *Chem. Sci.*, **2023**,14, 8878-8888, subjected to column chromatography and stored either as solids or toluene stock solutions in the dark at -20°C under N_2 atmosphere. Trioctylphosphine oxide (**1**), 4-bromo-2-(trifluoromethyl)phenol (**2**), 2-nitrobenzaldehyde (**3**), 4-*tert*-butylaniline (**4**) were purchased form Merck as technical grade and purified through column chromatography to obtain a suitable purity for the kinetics experiments. Column chromatography was carried out manually on Macherey-Nagel silica gel 60 (70-230 mesh). All NMR spectroscopy experiments were carried out on a Bruker AVIII 400 or 500 MHz AVIII HD Smart Probe, using the solvent residual signal as the internal standard. All chemical shifts (δ) are reported in ppm.

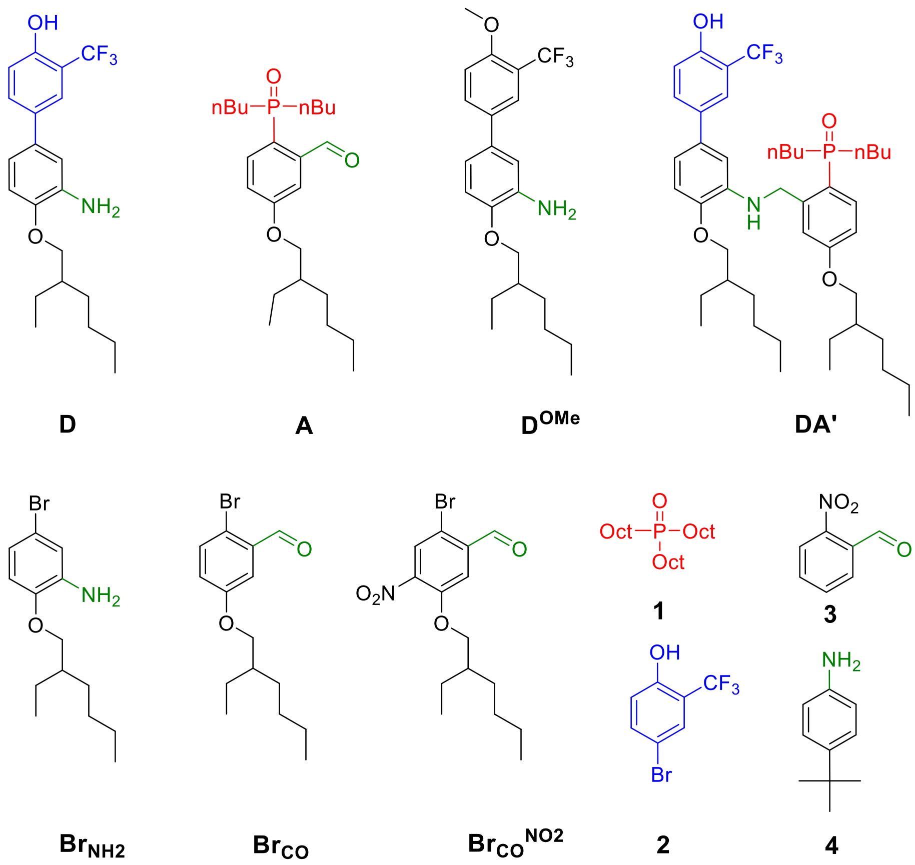

Scheme S1.1. Chemical structure of the molecules used in the study with the recognition units highlighted in blue (donor) and red (acceptor). The units involved in the imine chemistry are highlighted in green.

1.1 Setup of a ^{1}H NMR kinetics

For a classic ^{1}H NMR kinetics experiment, two vials were prepared as follows:

-**VIAL A**: containing **A**, **DA** or **1**.

-**VIAL B**: containing **D** or $\mathbf{D^{OMe}}$.

Just before preparing the NMR tube, the compounds in **VIAL A** were rapidly dissolved in 650 µL of chloroform-*d* that had been freshly filtered on basic alumina. Then, 600 µL of this solution were used to dissolve the compounds from **VIAL B** and then transferred in a screw-cap 5 mm NMR tube, so that all the compounds would have the desired concentration. The solution mixing time was set as time 0 of the kinetics run. The tube was tightly sealed also using PTFE tape to minimize the evaporation of solvent over time. The integrals of the imine were referred to the aldehyde signal for the calculations.

1.2 Synthetic procedures

Scheme S1.2 reports the synthesis scheme for the molecules used in the paper. For the intermediates **5**, **6**, **7**, **8**, **9**, we followed the exact procedures reported in Rosa-Gastaldo et al. *Chem. Sci.*, **2023**,14, 8878-8888. The NMR and mass characterizations matched the one reported therein. The new yields are reported in Scheme S1.2

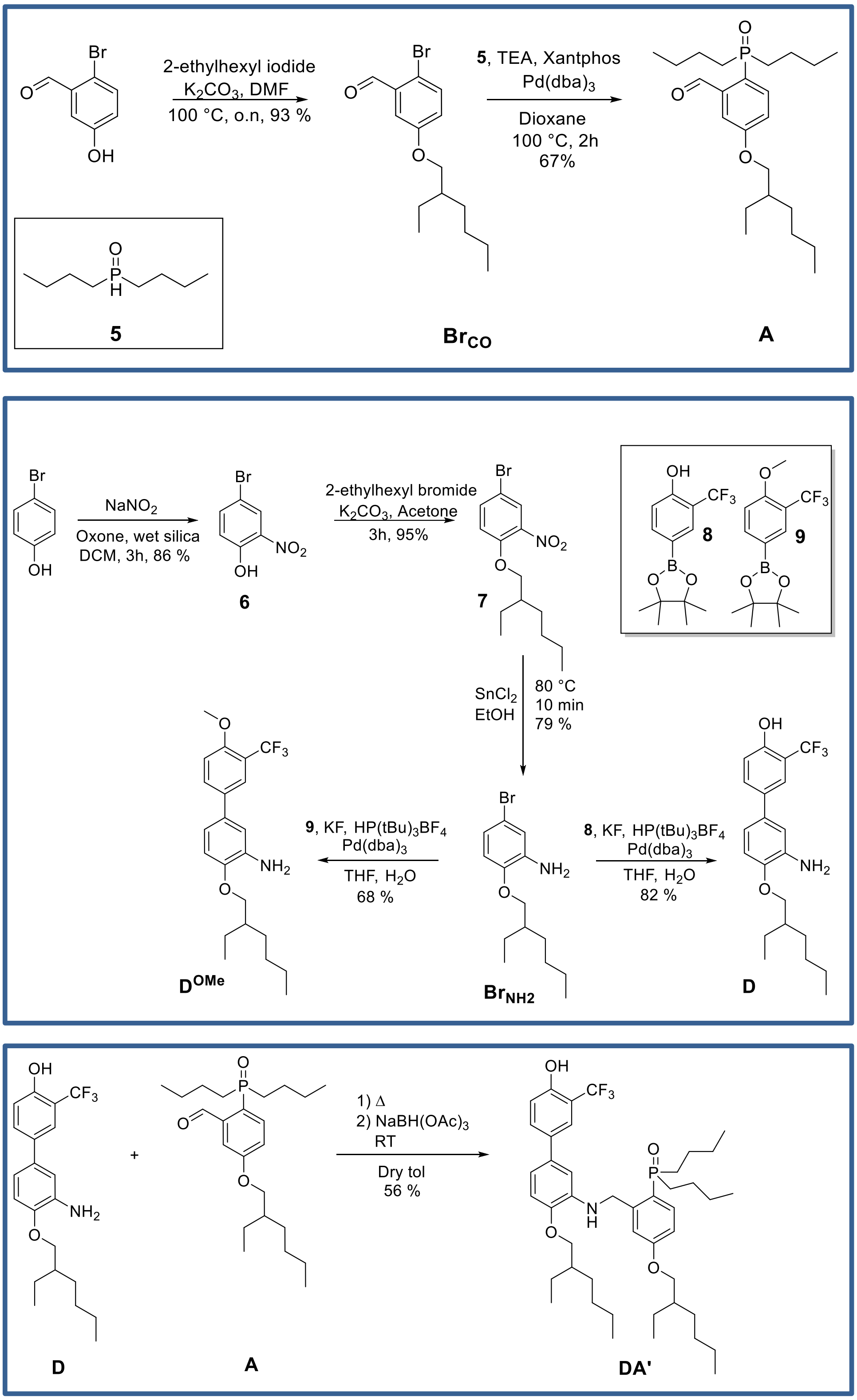

Scheme S1.2. Reaction pathways followed for the synthesis of the molecules reported in the paper.

A

Compound **2** (0.75 g, 2.39 mmol, 1 equiv) was mixed with the phosphine oxide **3** (790 mg, 4.87 mmol, 2 equiv), Xantphos (191 mg, 0.33 mmol, 0.14 equiv) and Pd_2dba_3 (300 mg, 0.33 mmol, 0.14 equiv); finally, previously degassed dioxane (10 mL, N_2 bubbling for 15 min) and triethylamine (751 mg, 1034 µl, 7.42 mmol, 3.1 equiv) were added, and the solution was stirred under nitrogen atmosphere, in dark conditions, for 2 h. The rection mixture was extracted with brine/EtOAc (4x20 ml), the organic fractions were collected, dried over Na_2SO_4 and the solvent removed in vacuum. The crude was purified through flash column chromatography EtOAc:MeOH (gradient of MeOH from 0% to 3%) to obtain the pure **A** as colourless sticky oil (632 mg, 67% yield). The mass and NMR data matched the one reported in the above-mentioned paper.

D

In a sealed tube compound **6** (503 mg, 1.68 mmol, 1.2 equiv), compound **7** (403 mg, 1.40 mmol, 1 equiv), KF (400 mg, 6.89 mmol, 4.9 equiv) and $HP(tBu)_3BF_4$ (12 mg, 0.041 mmol, 0.03 equiv) were suspended in freshly degassed (N_2 bubbling, 15 min) THF (3 mL) and H_2O (1 mL). Then $Pd_2(dba)_3$ (21 mg, 0.023 mmol, 0.016 equiv) was added and the reaction was stirred under N_2 atmosphere at 80°C for 3 h. Then it was extracted in brine/EtOAc (3x20 ml), the organic phases were collected, dried with $MgSO_4$, concentrated under vacuum and purified by flash column chromatography with PE:EtOAc (from 9:1 to 8:2) giving the pure desired monomer **D** as pale yellow solid (438 mg, 82% yield). The mass and NMR data matched the one reported in the above-mentioned paper.

D^{OMe}

In a sealed tube compound **6** (520 mg, 1.73 mmol, 1.2 equiv), compound **8** (435 mg, 1.44 mmol, 1 equiv), KF (370 mg, 6.37 mmol, 4.4 equiv) and $HP(tBu)_3BF_4$ (13 mg, 0.045 mmol, 0.031 equiv) were suspended in freshly degassed (N_2 bubbling, 15 min) THF (3 mL) and H_2O (1 mL). Then $Pd_2(dba)_3$ (24 mg, 0.026 mmol, 0.018 equiv) was added and the reaction was stirred under N_2 atmosphere at 80°C for 3 h. Then it was extracted in brine/EtOAc (3x20 ml), the organic phases were collected, dried with $MgSO_4$, concentrated under vacuum and purified by flash column chromatography with PE:EtOAc (from 9:1 to 8:2) giving the pure desired monomer **D^{OMe}** as pale yellow solid (387 mg, 68% yield). The mass and NMR data matched the one reported in the above-mentioned paper.

DA’

DA’ was synthesized via reductive amination. Monomers **A** (51 mg, 0.129 mmol, 1 equiv) and **D** (98 mg, 0.257 mmol, 2 equiv) were dissolved in dry toluene (5 mL) in a 10 ml flask at 110°C using a dean stark apparatus overnight. Then, $NaBH(OAc)_3$ (145 mg, 0.684 mmol, 5.3 equiv) was added and the mixture was stirred again until complete reduction of the imine (confirmed by ^{1}H-NMR). Then, a saturated solution of NaCl was added and the mixture was stirred until complete solubilisation. Hence, the organic phase was extracted, and the aqueous phase was washed (3x 20 ml) with EtOAc. The organic layers were collected and dried over Na_2SO_4, the solvent was evaporated, and the residue was purified via flash chromatography (gradient EP: EtOAc 5:5 to 3:7), giving the desired dimer as pale-yellow oil (57 mg, 56% yield). The mass and NMR data matched the one reported in the above-mentioned paper.

2. Complete ¹H NMR kinetics data

2.1 D 50 mM + A 50 mM

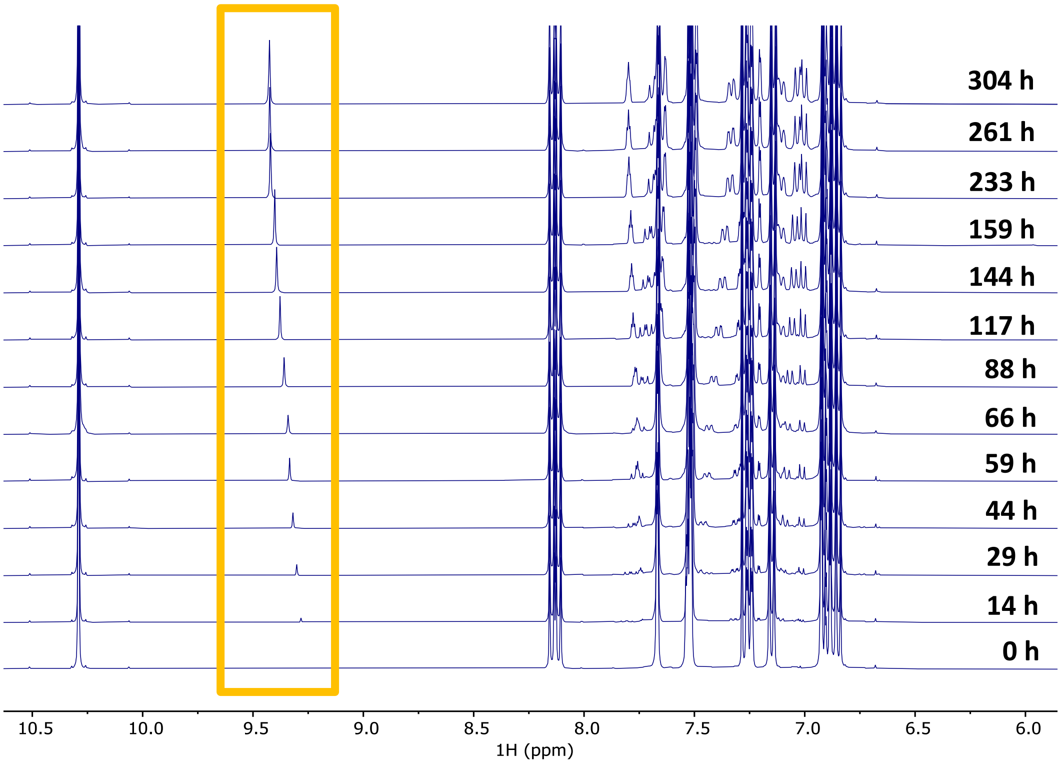

Figure S2.1. ¹H NMR spectra evolution of a solution containing **D** and **A** 50 mM in chloroform-*d* monitored until equilibrium was reached. The integrated data from this run have been used for the graph of Figure 3a of the manuscript. The orange frame highlights the imine signal region.

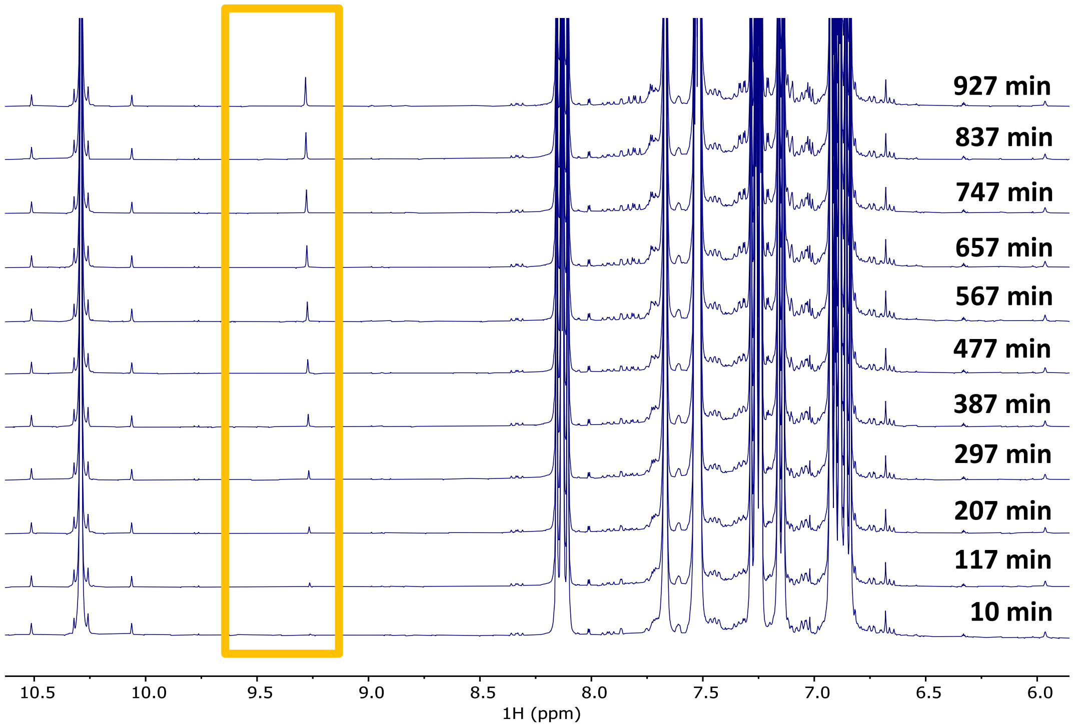

Figure S2.2. ¹H NMR initial spectra evolution of a solution containing **D** and **A** 50 mM in chloroform-*d*. The integrated data from this run have been used for the green trace in the graph of Figure 3b of the manuscript. The orange frame highlights the imine signal region.

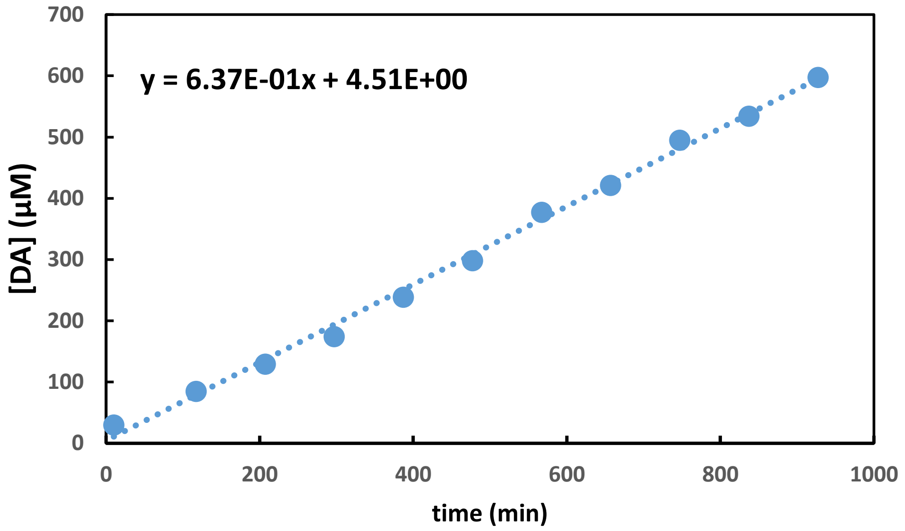

Figure S2.3. Best fit of the initial speed of the **DA** formation. Integrated data from the kinetics in Figure S2.2. V_{init} = 0.64 µM min^{-1}.

2.2 D^{OMe} 50 mM + A 50 mM

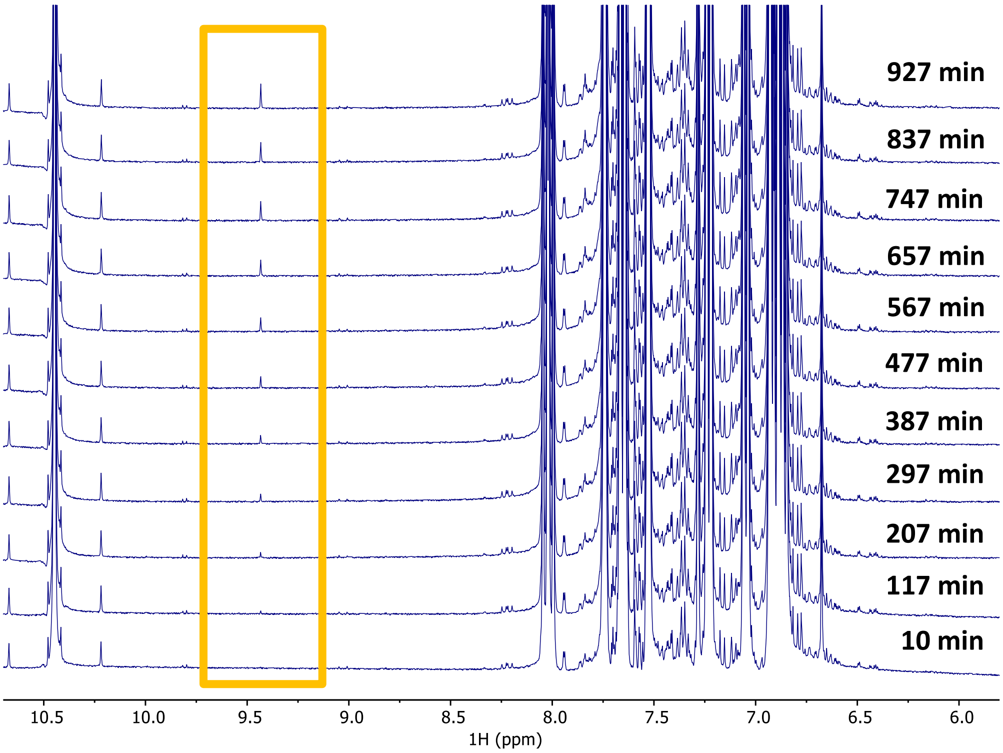

Figure S2.4. ^{1}H NMR spectra evolution of a solution containing **D^{Ome}** and **A** 50 mM in chloroform-*d*. The integrated data from this run have been used for the red trace in the graph of Figure 3b of the manuscript. The orange frame highlights the imine signal region.

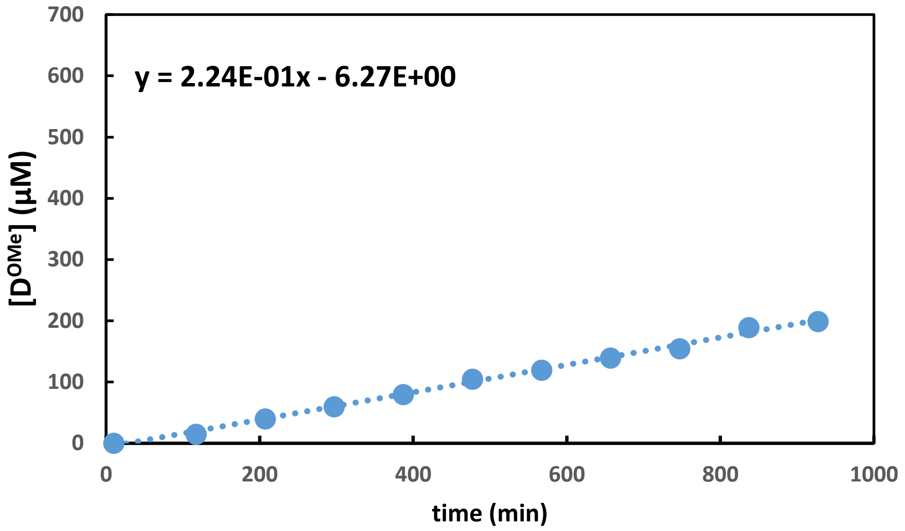

Figure S2.5. Best fit of the initial speed of the $\mathbf{D^{OMe}A}$ formation. Integrated data from the kinetics in Figure S2.4. V_{init} = 0.22 µM min^{-1}.

2.3 D 50 mM + A 50 mM + 1 50 mM

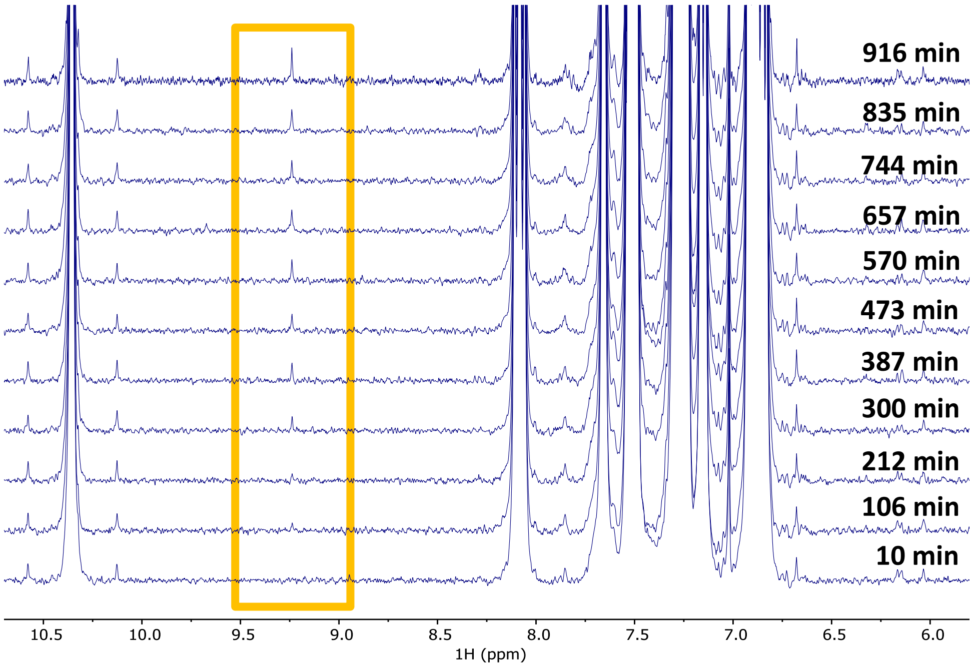

Figure S2.6. ^{1}H NMR spectra evolution of a solution containing **D**, **A** and **1** 50 mM in chloroform-*d*. The orange frame highlights the imine signal region.

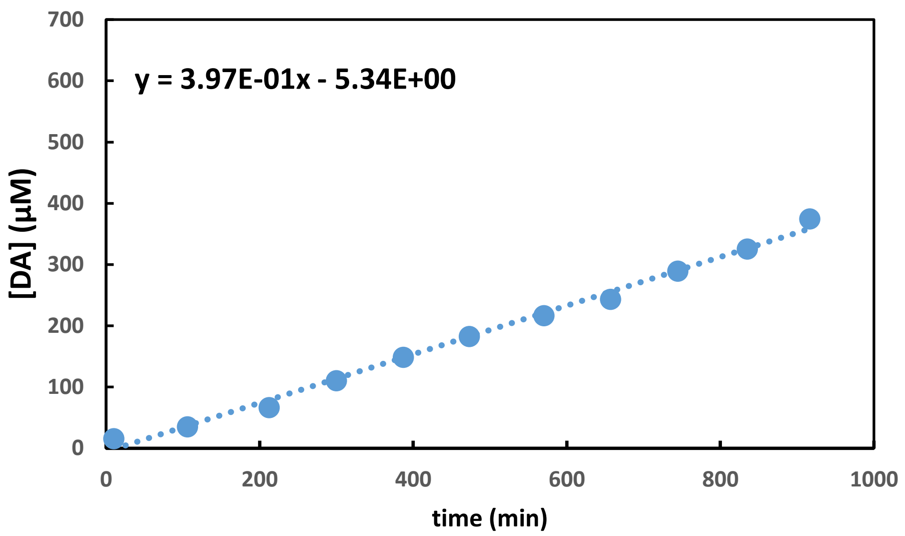

Figure S2.7. Best fit of the initial speed of the **DA** formation in presence of 50 mM of **1**. Integrated data from the kinetics in Figure S2.6. V_{init} = 0.40 µM min^{-1}.

2.4 D 50 mM + A 50 mM + DA' variable concentrations

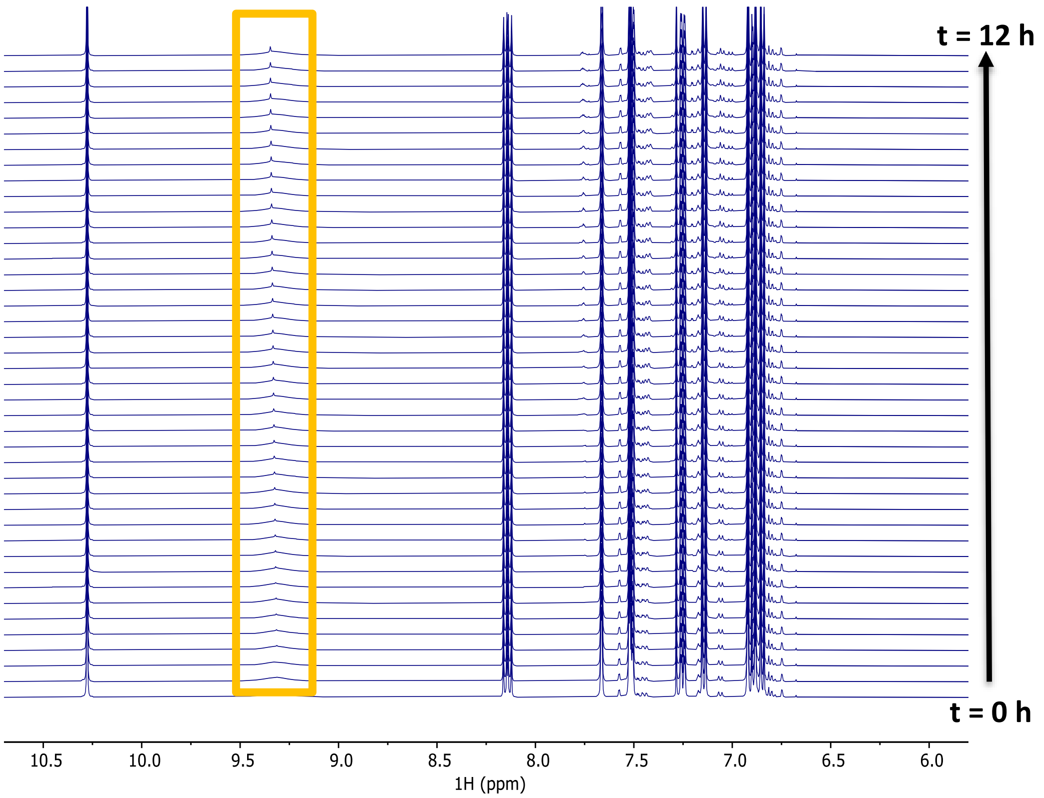

Figure S2.8. ^{1}H NMR spectra evolution of a solution containing **D**, **A** 50 mM and **DA'** 5 mM (0.1 equivalents) in chloroform-*d*. The reaction was monitored for 12 h. The integrated intensities of this run have been used in the graph of Figure 7a. (initial speed calculated in the first 300 minutes). The orange frame highlights the imine signal region.

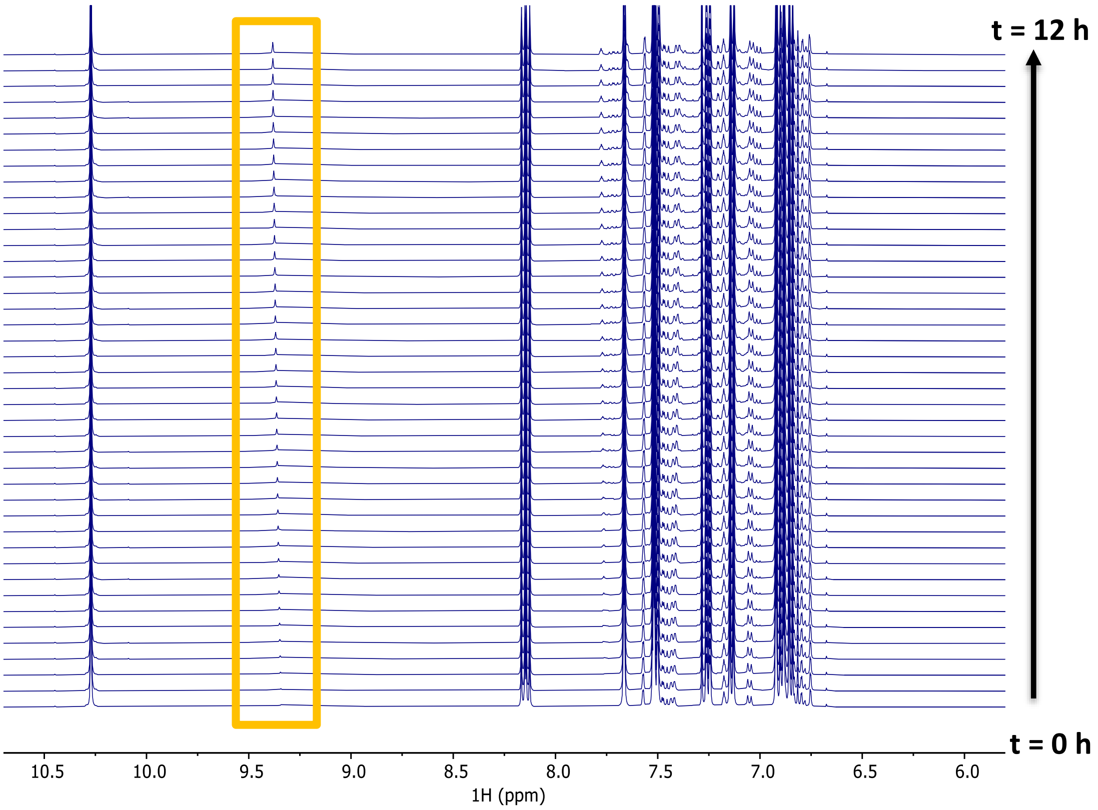

Figure S2.9 ^{1}H NMR spectra evolution of a solution containing **D**, **A** 50 mM and **DA'** 10 mM (0.2 equivalents) in chloroform-*d*. The reaction was monitored for 12 h. The integrated intensities of this run have been used in the graph of Figure 7a. (initial speed calculated in the first 300 minutes). The orange frame highlights the imine signal region.

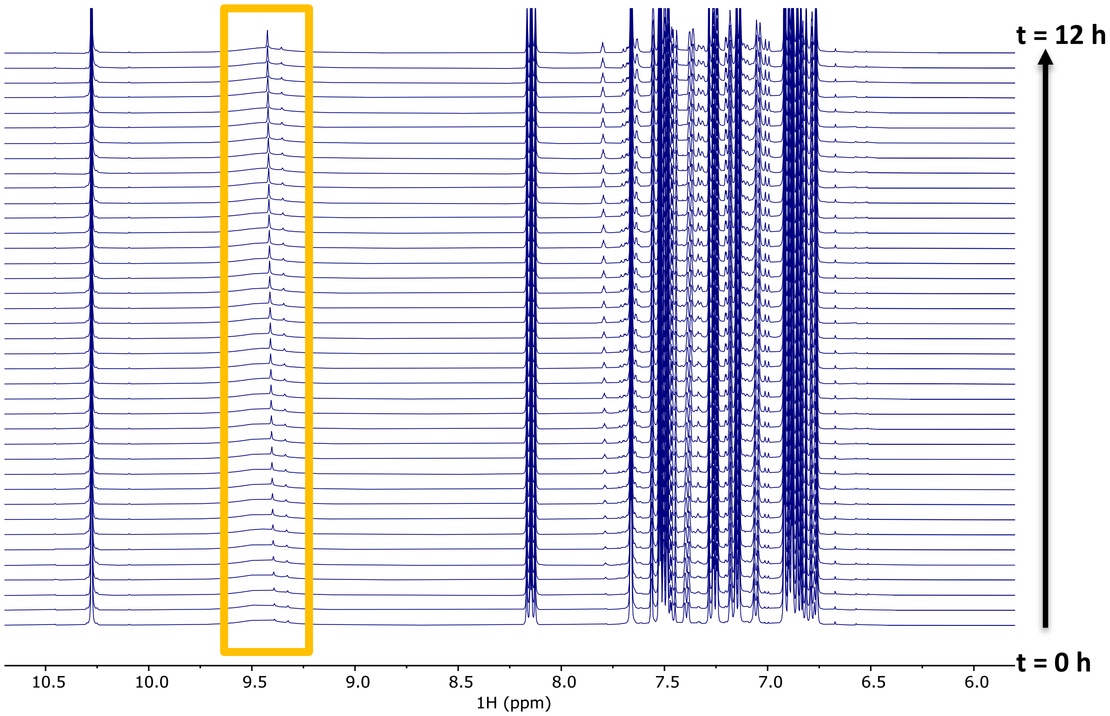

Figure S2.10. ^{1}H NMR spectra evolution of a solution containing **D**, **A** 50 mM and **DA'** 20 mM (0.4 equivalents) in chloroform-*d*. The reaction was monitored for 12 h. The integrated intensities of this run have been used in the graph of Figure 7a. (initial speed calculated in the first 300 minutes). The orange frame highlights the imine signal region.

DA’ equivalents	0.0	0.1	0.2	0.4
V_{init} (μM min^{-1})	0.53	3.50	4.80	5.60
error (μM min^{-1})	0.08	0.06	0.05	0.04

Table S1 V_{init} complete data (Figure 7b of the manuscript). Each kinetics was repeated twice, and the average value is reported with errors at the 95% confidence limit.

3. Evaluation of the phenol role in the catalysis of the imine formation

3.1 Br_{CO} 50 mM + Br_{NH2} 50 mM + 1 50 mM

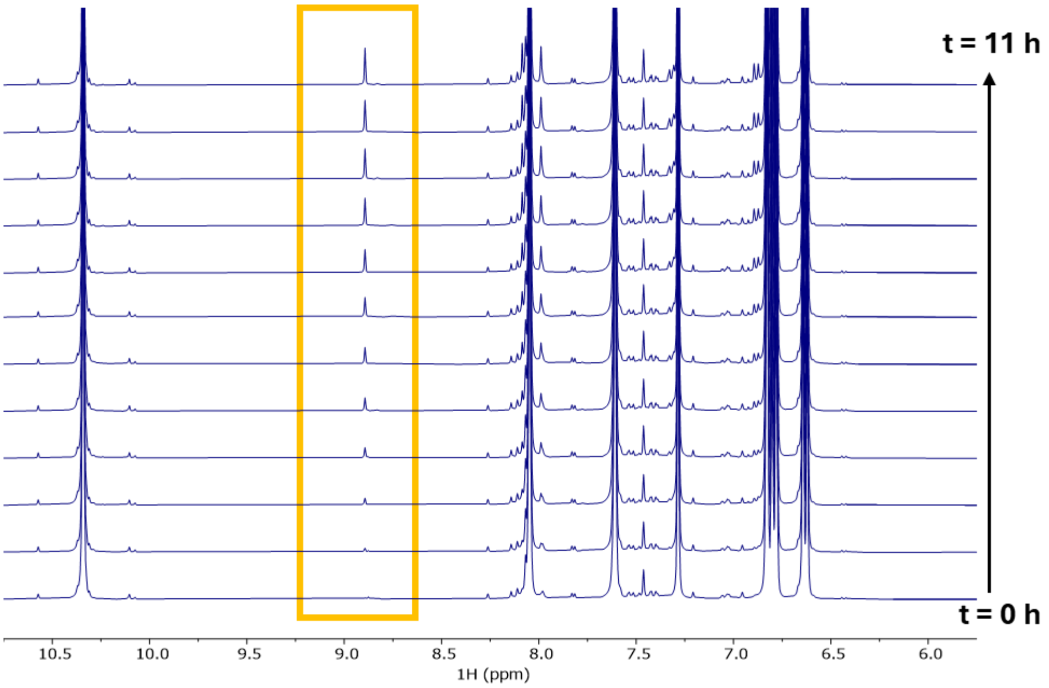

Figure S3.1. ^{1}H NMR spectra evolution of a solution containing **Br_{CO}**, **Br_{NH2}**, **1** all 50 mM in chloroform-*d*. The reaction was monitored for 11 h. The initial speed was calculated using the spectra obtained in the first 300 minutes. The orange frame highlights the imine signal region.

3.2 Br_{CO} 50 mM + Br_{NH2} 50 mM + 1 50 mM + 2 50mM

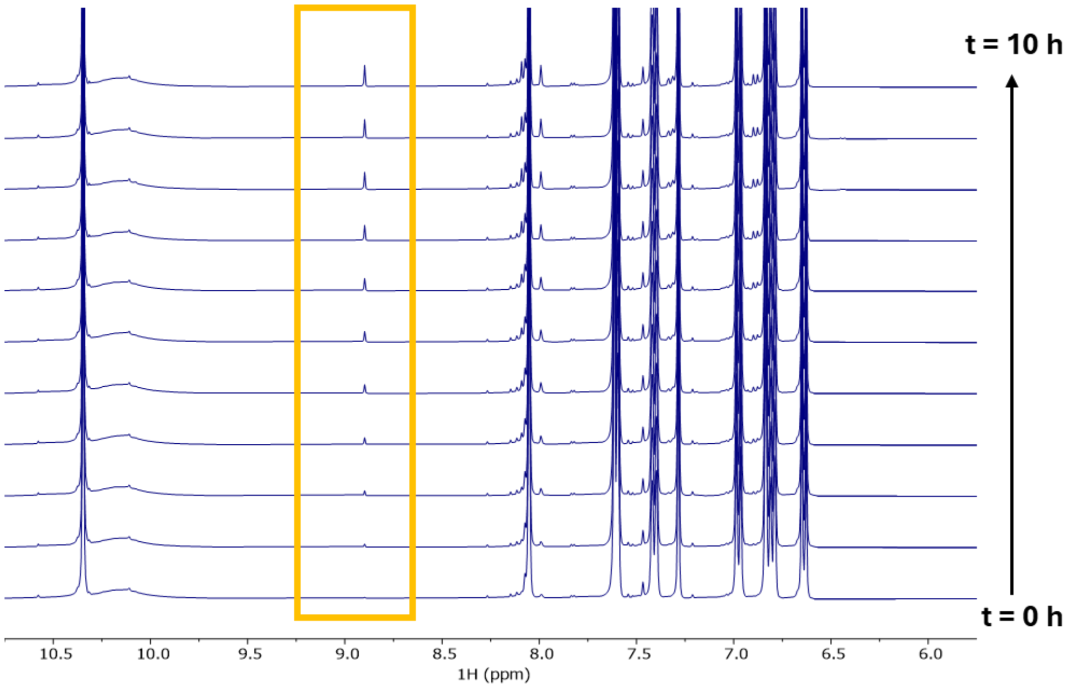

Figure S3.2. ^{1}H NMR spectra evolution of a solution containing **Br_{CO}**, **Br_{NH2}**, **1** and the phenol **2** all 50 mM in chloroform-*d*. The reaction was monitored for 10 h. The initial speed was calculated using the spectra obtained in the first 300 minutes. The orange frame highlights the imine signal region.

3.3 Br$_{CO}^{NO2}$ 50 mM + Br$_{NH2}$ 50 mM + 1 50 mM

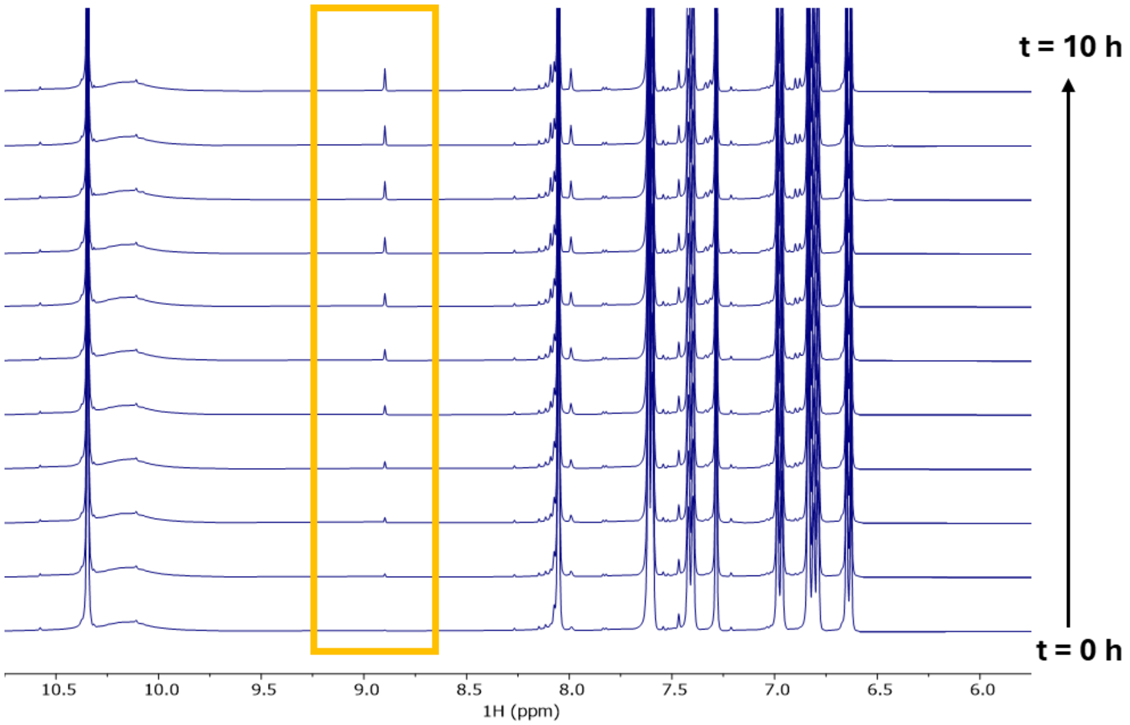

Figure S3.3. ^{1}H NMR spectra evolution of a solution containing **Br$_{CO}^{NO2}$**, **Br$_{NH2}$**, **1** all 50 mM in chloroform-*d*. The reaction was monitored for 10 h. The initial speed was calculated using the spectra obtained in the first 300 minutes. The orange frame highlights the imine signal region.

3.4 Br$_{CO}^{NO2}$ 50 mM + Br$_{NH2}$ 50 mM + 1 50 mM + 2 50 mM

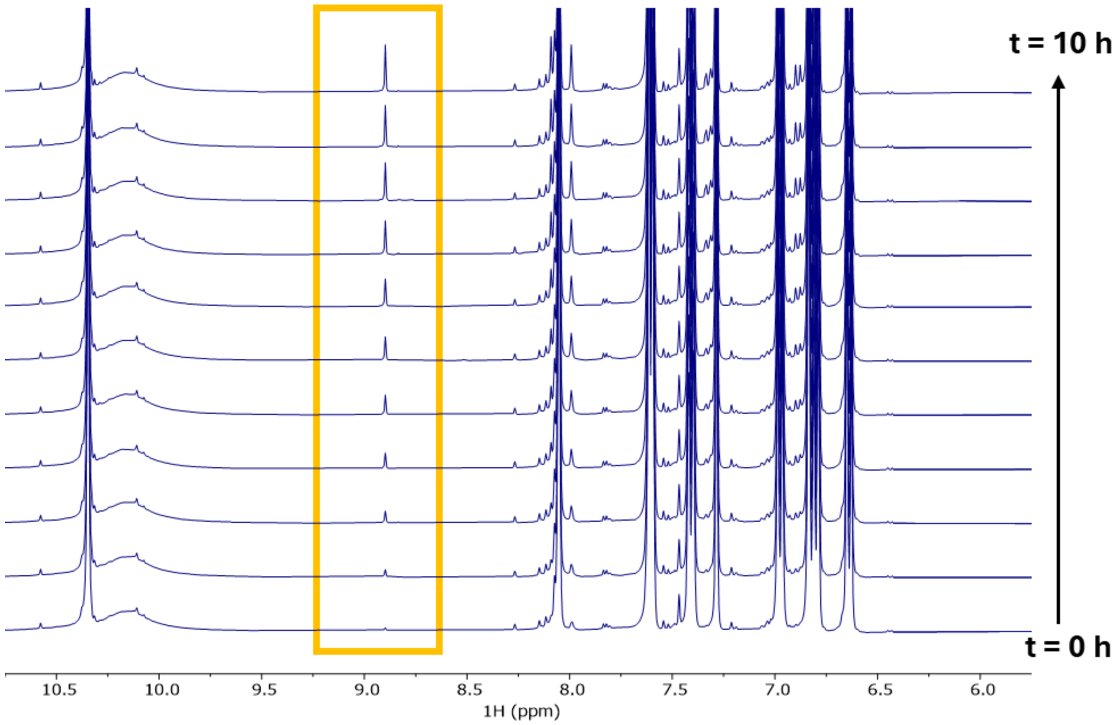

Figure S3.4. ^{1}H NMR spectra evolution of a solution containing **Br$_{CO}^{NO2}$**, **Br$_{NH2}$**, **1** and the phenol **2** all 50 mM in chloroform-*d*. The reaction was monitored for 10 h. The initial speed was calculated using the spectra obtained in the first 300 minutes. The orange frame highlights the imine signal region.

3.5 3 50 mM + 4 50 mM + 1 50 mM

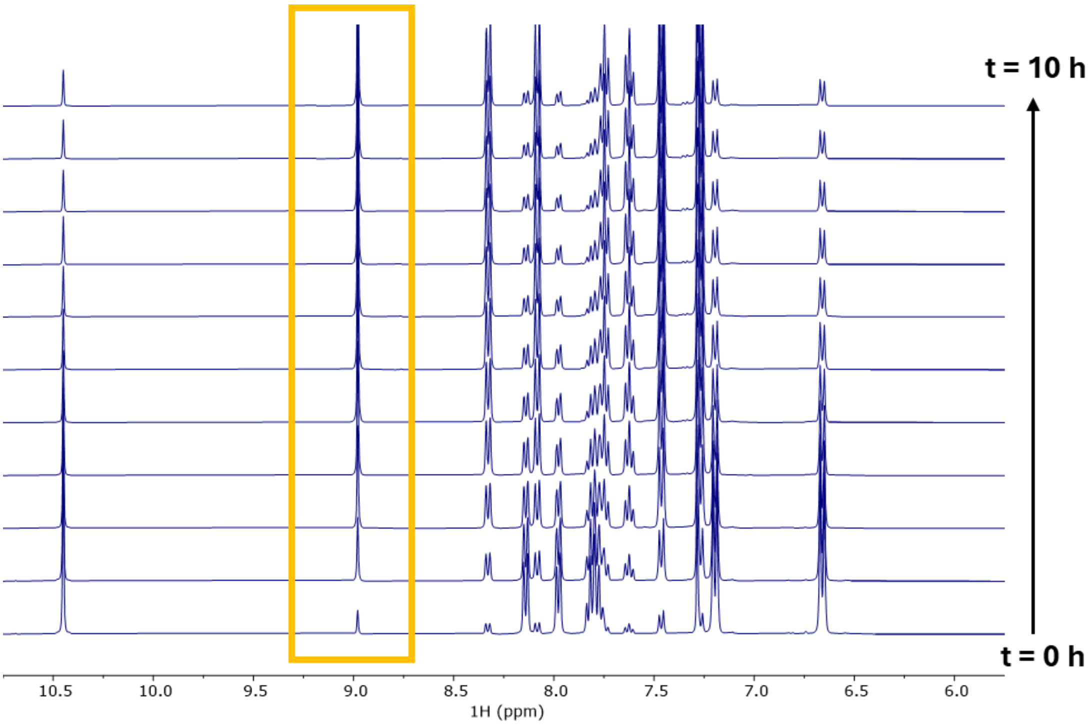

Figure S3.5. ^{1}H NMR spectra evolution of a solution containing **3**, **4**, **1** all 50 mM in chloroform-*d*. The reaction was monitored for 10 h. The initial speed was calculated using the spectra obtained in the first 300 minutes. The orange frame highlights the imine signal region.

3.6 3 50 mM + 4 50 mM + 1 50 mM + 2 50 mM

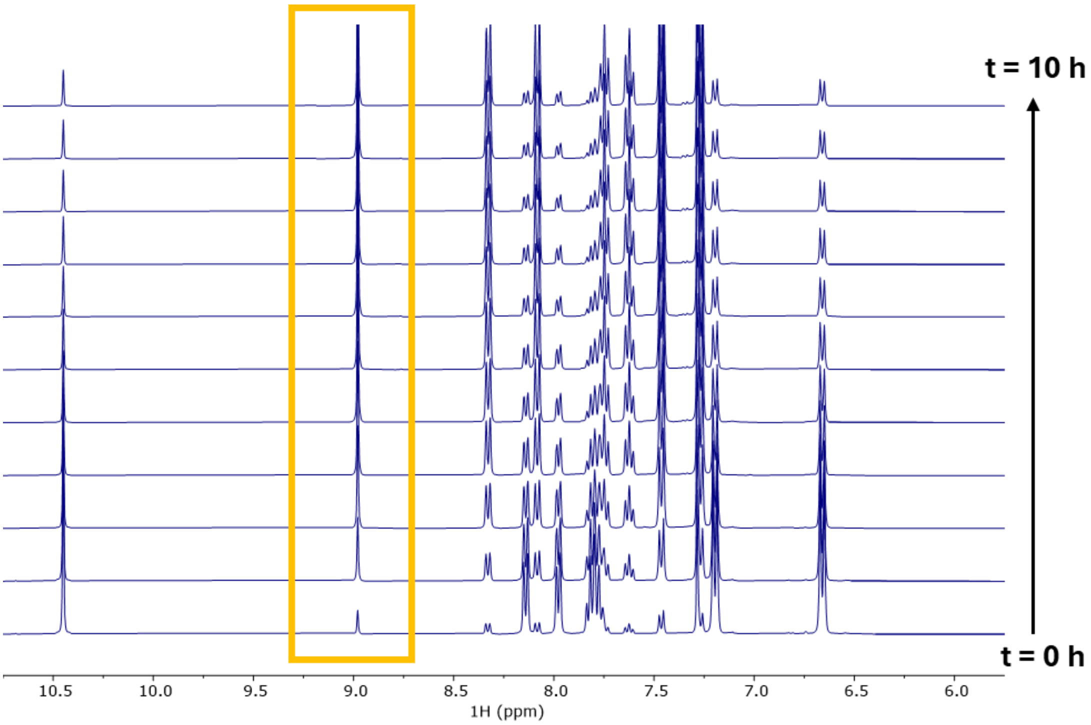

Figure S3.6. ^{1}H NMR spectra evolution of a solution containing **3**, **4**, **1** and the phenol **2** all 50 mM in chloroform-*d*. The reaction was monitored for 10 h. The initial speed was calculated using the spectra obtained in the first 300 minutes. The orange frame highlights the imine signal region.

3.7 A 50 mM + $BrNH_2$ 50 mM

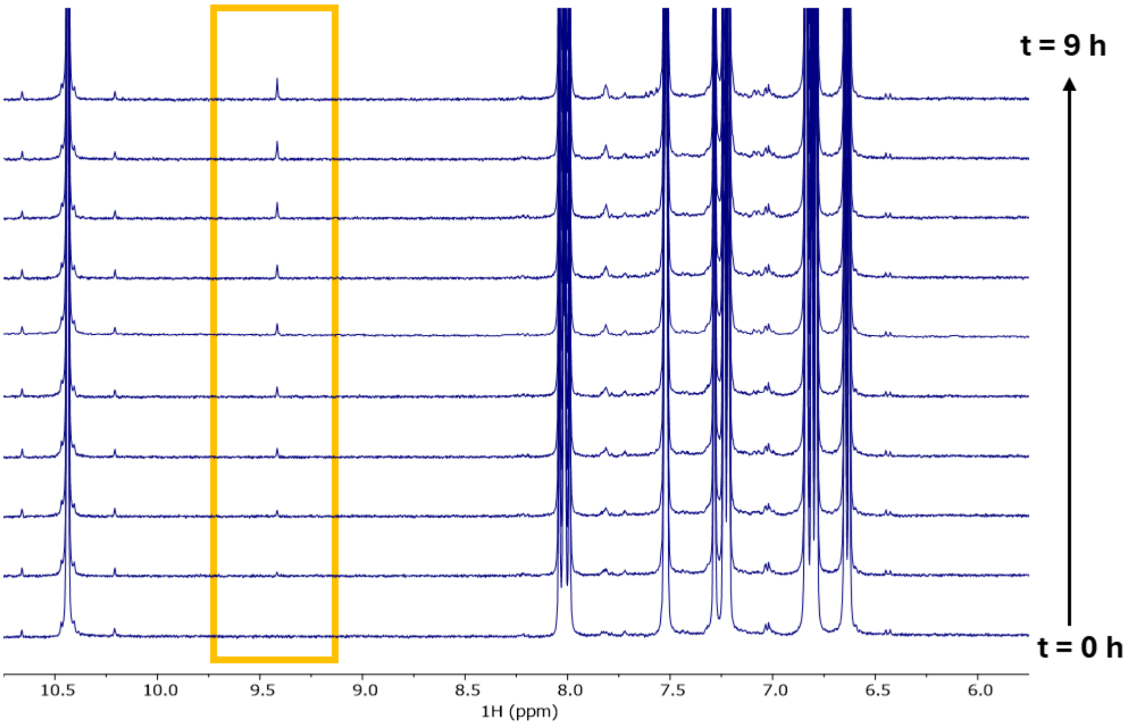

Figure S3.7. ^{1}H NMR spectra evolution of a solution containing **A**, **$BrNH_2$** all 50 mM in chloroform-*d*. The reaction was monitored for 9 h. The initial speed was calculated using the spectra obtained in the first 300 minutes. The orange frame highlights the imine signal region.

3.8 A 50 mM + $BrNH_2$ 50 mM + 2 50 mM

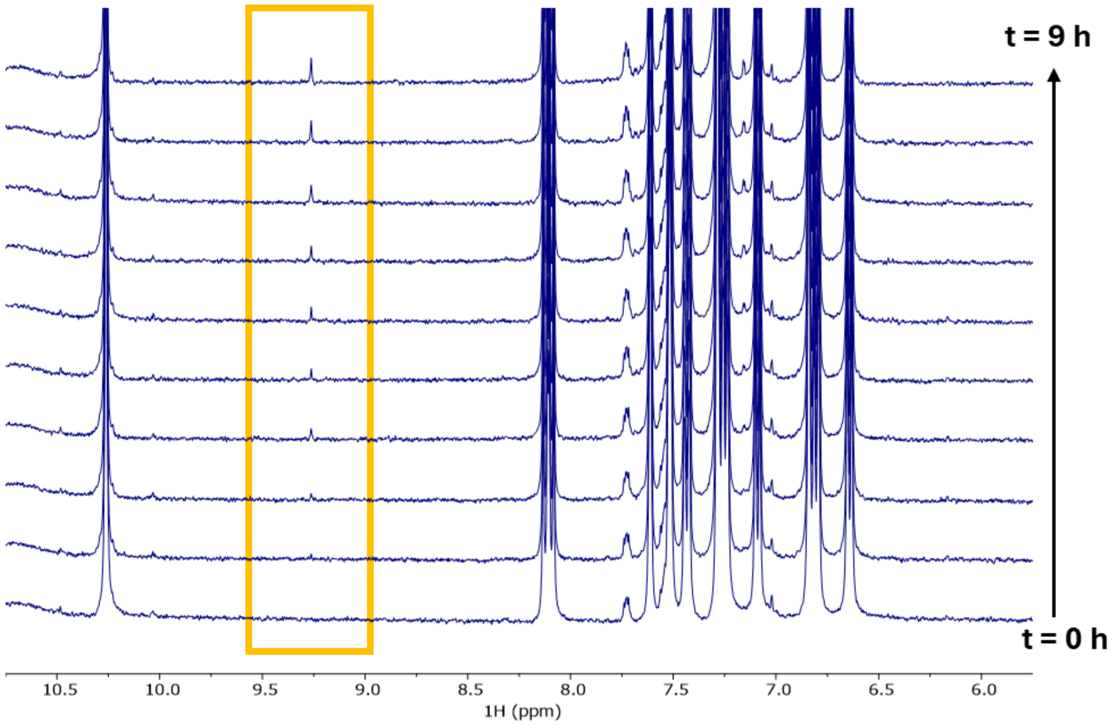

Figure S3.8. ^{1}H NMR spectra evolution of a solution containing **A**, **$BrNH_2$** and the phenol **2** all 50 mM in chloroform-*d*. The reaction was monitored for 9 h. The initial speed was calculated using the spectra obtained in the first 300 minutes. The orange frame highlights the imine signal region.

4. Competitive experiment

4.1 Competitive experiment

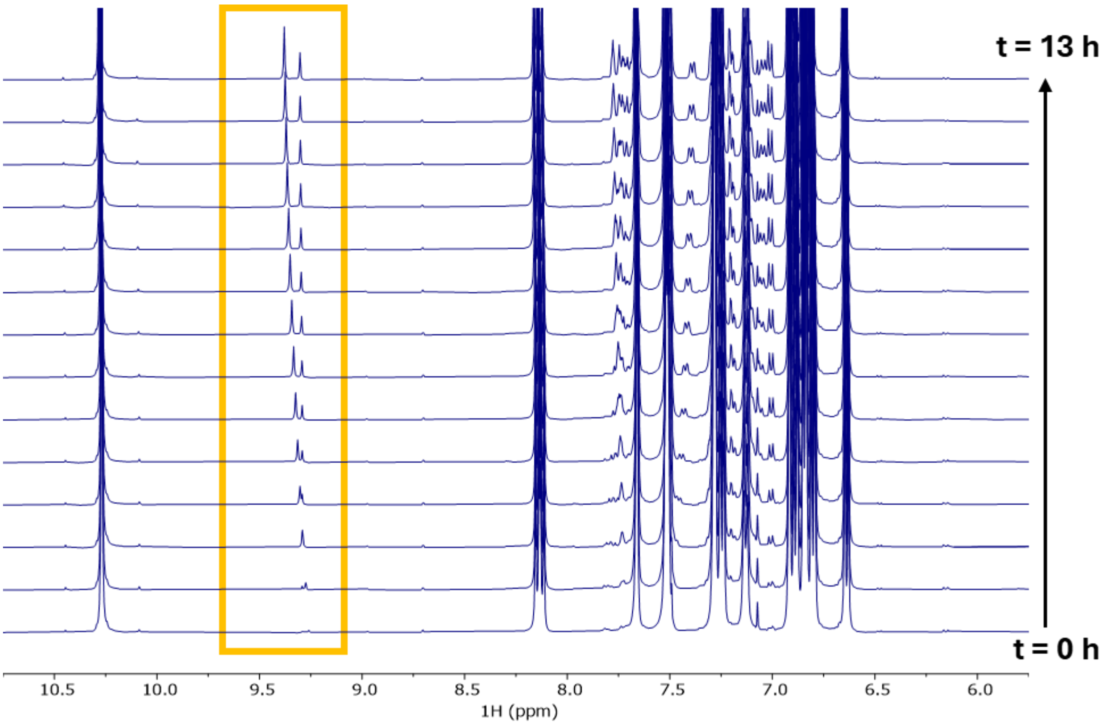

Figure S4.1. ^{1}H NMR spectra evolution of a solution containing **D**, **A** and **BrNH$_2$** 50 mM in chloroform-*d*. The reaction was monitored for 13 h. The integrated intensities of this run have been used in the graph of Figure 6a. (initial speed calculated in the first 300 minutes). The orange frame highlights the imine signal region.

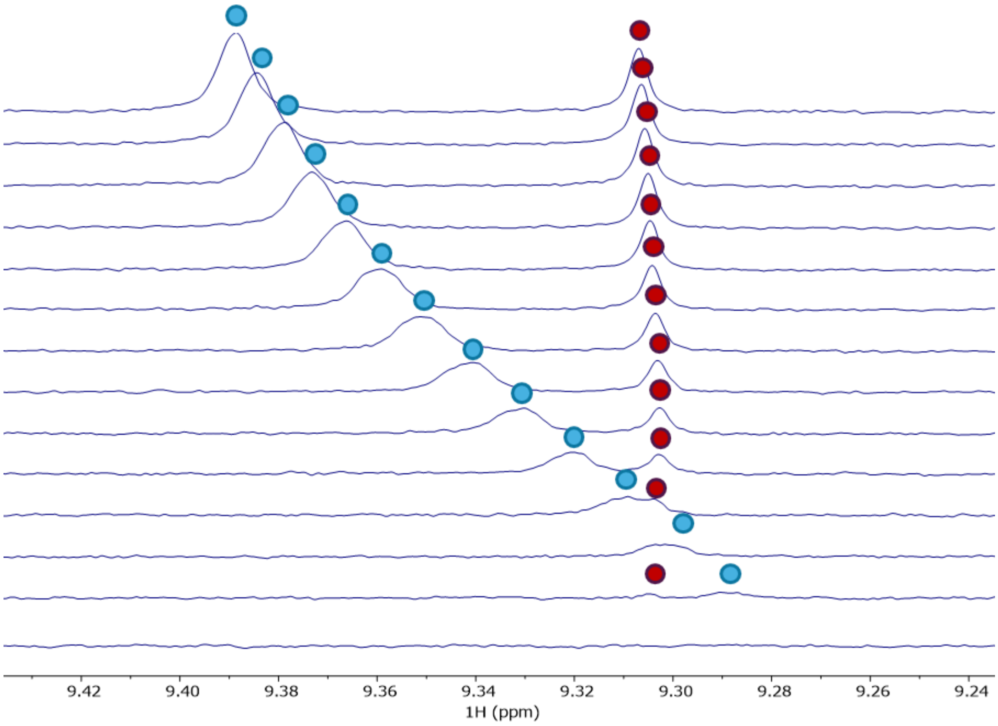

Figure S4.2. Detail of the imine region of the experiment reported in figure S4.1 of a solution containing **D**, **A** and **BrNH$_2$** 50 mM in chloroform-*d*. Blue circles indicate the signals of the **DA** imine, red circles the signals of **Br$_{NH2}$A** imine The reaction was monitored for 12 h. The integrated intensities of this run have been used in the graph of Figure 6a. (initial speed calculated in the first 300 minutes).
